# The utility of psychological readiness scales in predicting return to sport: a systematic review

**DOI:** 10.1186/s40359-025-03378-5

**Published:** 2025-11-03

**Authors:** Siqi Liu, Young-Eun Noh

**Affiliations:** Faculty of Sports and Exercise Science, Universiti Malaya, Kuala Lumpur, 50603 Malaysia

**Keywords:** Return to play, Psychological rehabilitation, Injured athlete, Recovery outcome

## Abstract

**Objective:**

This review aims to synthesise evidence on the predictive role of self-reported psychological readiness measures in return to sport (RTS) after sports injury.

**Methods:**

The study adhered to the Preferred Reporting Items for Systematic Reviews and Meta-Analyses (PRISMA 2020) statement, employing both electronic database searches (including Web of Science, Psychology & Behavioral Sciences Collection, PubMed, SPORTDiscus, and Scopus) and manual searches. The inclusion criteria for this study comprised two key elements: (1) articles published in international journals in English; (2) articles utilising psychological readiness for RTS scales in their research. The exclusion criteria included the following: (1) articles unrelated to sports injury topics; (2) articles about concussions; (3) psychological readiness unrelated to RTS; (4) grey literature; (5) review studies; (6) articles utilising psychological readiness for RTS scales that were non-English translations; and (7) articles examined mental states after sport injuries using only one type of measure, either emotional or self-efficacy rather than a comprehensive assessment. The methodological quality of the included studies was assessed using the Mixed Methods Appraisal Tool.

**Results:**

Sixty-two relevant studies were included. The results of the systematic review indicated that psychological readiness self-assessments can be categorised into four key domains: 1) predicting physical recovery outcomes (*n* = 24), 2) predicting return to sport practices (*n* = 18), 3) predicting quality of life-related to injury and reinjury rates (*n* = 7), and 4) predicting post-return sport performance levels and physical activity levels (*n* = 13).

**Conclusion:**

This review underscores the value of psychological readiness assessments as predictors of RTS outcomes. The findings support their clinical utility, while also highlighting the need for further research into injury-specific and objective measures to enhance assessment accuracy. This systematic review has been registered in PROSPERO (registration number: CRD42025642947).

After experiencing a sports injury, some athletes permanently leave their athletic careers. For others, the recovery process takes a different path, often marked by challenges and uncertainties [1]. However, many athletes successfully recover and return to sport (RTS) after an extended period of rehabilitation [2]. This process of recovery is complex and can be conceptualised through various stages [3]. At the first International Return to Sport Congress, 17 expert clinicians defined RTS as a continuum consisting of three elements: return to participation, return to sport, and return to performance [3]. They also agreed that RTS should be considered an integrated process involving both physical and psychological recovery. This is because sports injuries not only result in significant declines in physical function and quality of life but also negatively impact athletes’ mental health [1, 2]. For example, after sports injuries, athletes may experience negative emotions, such as fear of reinjury, anger, frustration, depression, anxiety, and shock [4–6]. These negative emotions can affect postoperative outcomes and impede psychological rehabilitation [7, 8]. 

Furthermore, psychological rehabilitation and physical recovery are not always synchronous [9, 10]. Generally, psychological rehabilitation lags behind physical recovery following a sports injury [11]. Therefore, even when an athlete is physically ready to return to training or competition, these negative emotions may persist throughout the RTS continuum [12, 13]. Consequently, Hurley et al. [14] argued that psychological challenges are a major reason for people’s inability to RTS. In an interview study, sports physical therapists also perceived psychological barriers in anterior cruciate ligament (ACL) injury patients to be more severe than physical issues [15].

Psychological readiness to return to sport after injury refers to the possession of (or access to) psychological resources that facilitate a safe, productive, and enjoyable return to sport—and to freedom from psychological attributes or states that impede such a return [16]. Recent perspectives view psychological readiness for RTS as a three-dimensional construct including cognitive appraisals, affective components, and behavioural components [17]. The affective components include negative emotions, such as anxiety about reinjury or movement. Thus, the persistence of negative emotions after a sports injury can also impact psychological readiness for RTS [18]. Moreover, lower levels of confidence can affect the cognitive appraisal dimension within psychological readiness for RTS [19, 20].

Impaired psychological readiness, which has been associated with negative emotions and low confidence, has also been associated with slower physical recovery (e.g., muscle recovery), greater joint laxity, and higher pain levels [21–23]. These psychological factors have further been linked to suboptimal performance and higher reinjury rates after return to sport [24, 25]. Certain avoidance behaviors, such as reduced adherence to rehabilitation, may also influence psychological readiness in injured athletes during the rehabilitation process and at the time of return [17].

Given the extensive impact of psychological readiness on the RTS continuum and outcomes, the consensus from multiple experts has advocated for the assessment of psychological readiness during the RTS continuum [26–29]. A practical method for assessing psychological readiness is through validated subjective questionnaires [30], such as the Anterior Cruciate Ligament–Return to Sport after Injury (ACL-RSI) scale [31], the Injury–Psychological Readiness to Return to Sport (I-PRRS) scale [32], the Superior Labrum Anterior–Posterior–Return to Sport after Injury (SLAP-RSI) scale [33], and the Shoulder Instability–Return to Sport after Injury (SI-RSI) scale [34]. These questionnaires are clinician-friendly and hold significant clinical value in evaluating an athlete’s readiness for RTS after a sports injury [17, 35, 36].

These measures can assist practitioners in making informed RTS decisions [37, 38] and predict poor recovery outcomes and secondary injury rates [39]. Furthermore, they provide a more comprehensive and accurate reflection of an individual’s mental state compared to tools that only assess emotions such as fear [40]. In addition, compared to physical assessments, psychological readiness assessments may more accurately predict athletes’ post-return performance and reinjury rates [41, 42].

However, while numerous psychological readiness assessments in RTS contexts exist [3, 17, 39, 43–59], their application in practice remains limited [60]. For example, a survey of 439 physical therapists found that only 19.1% used psychological questionnaires to inform RTS decisions [61]. To reduce the gap between research and practice, Evans and Brewer [62] recommended that researchers prioritize establishing the validity of psychological readiness measures to enhance practitioners’ trust and encourage their adoption. Predictive validity is particularly important, as these tools can support practitioners in making informed decisions about an athlete’s readiness to return to sport. However, the predictive utility of these measures remains unclear, which may undermine practitioners’ confidence in their application. A systematic review is an effective method for synthesising and integrating established evidence [63]. Therefore, this study conducted a systematic review to consolidate the evidence on the predictive role of self-assessment scales for psychological readiness in RTS. By synthesizing comprehensive evidence, we aimed to highlight the practical significance of psychological readiness assessments and promote the application of sports injury psychology research in clinical settings. The research question guiding this review was: What are the predictive roles of self-assessment scales for psychological readiness in RTS when applied in practice?

## Methodology

This systematic review has been registered in PROSPERO (registration number: CRD42025642947). This review followed the Preferred Reporting Items for Systematic Reviews and Meta-Analyses (PRISMA 2020) statement [63] and employed both electronic database searches and manual searches. The electronic database searches included Web of Science, Psychology & Behavioral Sciences Collection, PubMed, SPORTDiscus, and Scopus. The database searches used the following keywords to search titles, keywords, and abstracts: (“return to sport*” OR “return to performance*” OR “return to play”) AND (psycholog* OR biopsychosocial* OR psychosoc* OR appraisal OR expectation OR kinesiophobia OR self-efficacy OR “negative state*” OR fear* OR anxiety* OR stress* OR disorder* OR distress* OR depress* OR pressure* OR frustrat* OR anger* OR symptom* OR emotion* OR insecurity OR efficacy OR mood OR shock* OR confiden* OR motivation* OR readiness OR adherence OR “avoidance behavio*r*”). This search strategy encompassed the entire RTS continuum and employed truncation, thereby broadening the scope of the search. Keywords related to psychological readiness were obtained from existing related literature reviews [17, 47–49, 55, 59, 64, 65]. Since the first psychological readiness assessment scale for RTS was established in 2008 [31], the search date range was from 2008 to July 2024. The final search date was July 25, 2024. 

The manual searches involved both forward and backward citation tracking through the Google Scholar search engine, and the results were stored in Zotero (2016) for further screening decisions by the first author. The manual searches included (1) review articles, editorial commentary articles, and expert consensus articles [3, 43–59, 62]; (2) articles cited in the included studies identified via the electronic database searches; (3) articles about psychological readiness measurement tools (e.g., ACL-RSI scale [31], I-PRRS scale [32], and SI-RSI scale [34]).

The inclusion criteria for this study comprised two key elements: (1) articles published in international journals in English; (2) articles utilising psychological readiness for RTS scales in their research. The exclusion criteria included the following: (1) articles unrelated to sports injury topics, such as RTS after pandemics or post-pregnancy RTS; (2) articles about concussions, as concussions may affect the validity of self-assessment questionnaires due to cognitive impairments [3, 66] psychological readiness unrelated to RTS, such as pre-surgery psychological preparation or pre-competition psychological preparation; (4) grey literature; (5) review studies; (6) articles utilising psychological readiness for RTS scales that were non-English translations; and (7) article exclusively used the Tampa Scale for Kinesiophobia [67], the Re-Injury Anxiety Inventory [68], and the Knee–Self-Efficacy Scale [69] as these only reflect one aspect of psychological readiness (e.g., fear, anxiety, or self-efficacy) and cannot comprehensively reflect an individual’s psychological readiness for RTS [17, 40]. Therefore, this review excluded studies that used only these single emotional or self-efficacy measures to explore mental states after sports injuries.

The literature screening involved four steps: (1) duplicate removal, (2) primary screening, (3) secondary screening, and (4) expert consensus. The duplicate removal was carried out in Zotero (2016). The primary screening process included the independent assessment of titles and abstracts by the first and second authors. The secondary screening involved screening the included full-text literature based on the established inclusion and exclusion criteria. Any discrepancies in the inclusion outcomes during the primary and secondary screening processes were resolved through discussions between the two authors. Expert consensus was reached through deliberations among all the authors, engaging in discussions with an expert who has publication experience in international sport psychology journals. This collaborative process aimed to achieve a unanimous conclusion regarding the inclusion of articles in this review.

For the final included articles, the first and second authors independently extracted, analysed, and coded the data into two tables (see Tables 1 and 2). Table 1 presents the overall information of the included studies, such as the research aim(s), research design, and conclusion(s). Table 2 provides detailed information about the participants, sports injuries, and measurements used in the included studies. The categories of summarised information in Table 2 were designed based on summary tables from previous systematic reviews [23, 24, 47, 48, 57, 59, 65, 129, 130]. Additionally, the categories of Table 2 were continually updated during the extraction of the included literature and eventually included the authorship, publication year, participant information (i.e., population groups, types of sport, sports experience, number of participants, gender, age, and anthropometric measurements), injury information (i.e., treatment methods, time duration from injury to surgery, type of surgery, graft types, surgical history, injury context, and injury mechanism), and measurement information (i.e., measurement time, tools, and score). In addition, when the authors of the included studies indicated in their methodology sections that they had collected data relevant to our summarised topics (e.g., sports experience, time duration from injury to surgery, and timing of the psychological readiness measurement) but had not reported these data in their studies, the first author contacted them via email to request the original data, which were subsequently included in our Table 2. The first author verified the content of the summary table during the methodological quality appraisal. Any disputes were resolved through discussion between all the authors.

**Table 1:** Summary of aim(s), research design, and conclusion(s) from the included articles (Alphabetical Order by Domain)

Author(s) and Year	Aim(s)	Research Design	Conclusion(s)
**(1) Predicting Physical Recovery Outcomes**			
Aizawa et al., 2020 [70]	To identify factors that contribute to an athlete’s psychological readiness to return after ACLR to sports that require cutting, pivoting, and jump-landings.	Cross-sectional	Psychological readiness assessment at six months post-surgery can predict subjective running ability.
Aizawa et al., 2022 [71]	To identify specific physical functions related to the psychological readiness of patients aiming to RTS at 6 months after ACLR.	Cross-sectional	Psychological readiness assessment at 6 months post-surgery can predict the weight ratio of knee strength.
Baez et al., 2023 [72]	To examine the relationship between psychological readiness and the presence of persistent knee symptoms.	Cross-sectional	Psychological readiness assessment at eight months post-surgery can predict persistent knee symptoms.
Borawski et al., 2024 [73]	To explore whether psychological factors may contribute to reduced return to duty in military members.	Cross-sectional	Psychological readiness assessment at 18 months post-surgery can predict neuromuscular asymmetries.
Correa et al., 2023 [74]	To compare the performance in field tests, dynamic knee valgus, knee function, and kinesiophobia of soccer players who were psychologically ready and not ready to return to unrestricted training or competitions after ACLR.	Cross-sectional	Psychological readiness assessment at 7 months post-ACLR can predict knee valgus motion.
Cronstrom et al., 2023b [75]	To investigate the relationship between psychological readiness and physical function.	Cross-sectional	Psychological readiness assessment at 1 year post-surgery can predict single leg hop for distance.
Dombrowski et al., 2024 [76]	To assess the relationships between physical function tests of the operative limb and psychological readiness to RTS after ACLR by sex.	Cross-sectional	Psychological readiness assessment 6–8 months after ACLR can predict single-leg drop landing knee excursion.
Erickson et al., 2022 [77]	To investigate the relationship between psychological readiness and self‐reported functional outcomes, quadriceps strength, and knee mechanics.	Prospective cohort	Psychological readiness assessed at 3 months post-ACLR can predict knee function, knee-related quality of Life, and quadriceps strength Limb symmetry at 6 months.
Fones et al., 2020 [78]	To determine the relationship between psychological readiness and subjective knee evaluation.	Retrospective case–control	Psychological readiness assessment at 4 years post-surgery can predict subjective knee function evaluation outcomes.
Hart et al., 2020 [79]	To investigate the relationship between patient-reported and performance-based function.	Cross-sectional	Psychological readiness assessment at one-year post-surgery can predict patient-reported and performance-based function.
Högberg et al., 2023 [80]	To investigate the relationship between the eccentric NordBord test and the seated concentric Biodex test with patient-reported outcomes, during the first year of rehabilitation after ACLR.	Prospective cohort	Psychological readiness assessment at 8 months post-surgery can predict knee flexor absolute strength at 8 and 12 months.
Hurley et al., 2022a [81]	To evaluate factors associated with satisfaction and shoulder function.	Retrospective cohort	Psychological readiness assessment at 5 years post-surgery can predict postoperative satisfaction and subjective shoulder function.
Legnani et al., 2023 [82]	To investigate the relationships between and psychological readiness and jumping performance.	Prospective cohort	Psychological readiness assessment conducted at 6 months post-surgery can predict jumping ability.
Milewski et al., 2023 [83]	To prospectively compare differences in patients at 6 months after primary ACLR.	Prospective cohort	Psychological readiness assessment at 6 months post-surgery can predict subjective knee function evaluation outcomes.
Nagelli et al., 2019 [84]	To investigate the relationship between psychological readiness and single-leg landing biomechanics.	Cross-sectional	Psychological readiness assessment at 9 months post-ACLR can predict sagittal plane knee range of motion.
Peebles et al., 2021 [85]	To determine the relationship between psychological readiness and side-to-side symmetry during jump-landing.	Cross-sectional	Psychological readiness assessment at 6 months post-ACLR can predict asymmetrical landing dynamics and symmetry in joint extension torque.
Sanborn et al., 2022 [86]	To assess the impact of different surgical procedures on psychological readiness.	Randomized controlled trial	Psychological readiness assessment at 6 months after ACL surgery can predict hamstring and quadriceps strength.
Schilaty et al., 2023 [87]	To determine the motor control relationship between thigh musculature motor unit characteristics and psychological readiness to RTS between ACL injured and healthy controls.	Prospective cohort	Psychological readiness assessed 6 months after ACLR can predict the neuromuscular adaptation of athletes.
Sugarman et al., 2022 [88]	To investigate differences in functional performance and psychological readiness to RTS among athletes who have undergone primary ACLR.	Descriptive cohort	Psychological readiness assessment at 8 and 9 months after ACLR can predict knee flexion function.
Ueda et al., 2022 [89]	To examine the relationship between RTS criteria and psychological readiness.	Cross-sectional	Psychological readiness assessment at 12 months post-ACLR can predict hamstring strength and single-leg hop distance.
Webster and Feller, 2020 [90]	To test whether specific types of tests are associated with a return to competitive sport.	Prospective cohort	Psychological readiness assessment at 6 months post-ACLR surgery can predict self-reported measures of knee-related symptoms and function.
Webster et al., 2018 [91]	To identify factors that contribute to an athlete’s psychological readiness to RTS after ACLR.	Cross-sectional	Psychological readiness assessment at 12 months post-surgery can predict limb symmetry.
Zarzycki et al., 2018 [92]	To determine the relationship between kinematic and kinetic measures of knee symmetry during gait and psychological readiness to return to sport following ACLR.	Cross-sectional	The assessment of psychological readiness 6 months post-ACLR surgery can predict knee joint movement symmetry.
Zwolski et al., 2023 [93]	To determine the relationship between actual physical competence, perceived physical competence, and psychological readiness.	Cross-sectional	Psychological readiness assessment within 8 weeks of medical clearance to RTS can predict perceived personal competence.
**(2) Predicting Return to Sport (RTS) Practices**			
Aizawa et al., 2024 [94]	To investigate the relationship between physical function and ACL-RSI scores in patients after ACLR.	Cross-sectional	Psychological readiness assessment at 6 months post-surgery can predict the level of sports performance after RTS.
Beischer et al., 2019 [95]	To investigate the difference in psychological readiness between athletes returning to sport and those who have not returned post-ACLR.	Retrospective case–control	Psychological readiness assessment at 8 months post-surgery can predict RTS practices.
Bohu et al., 2021 [96]	To report the rate and time of RTS during the first 8 months following the Latarjet.	Retrospective cohort	Psychological readiness assessment at 8 months post-surgery can predict RTS practices.
Colasanti et al., 2023 [33]	To determine why athletes did not RTS following operative management of SLAP tears, compare these athletes to those who did RTS.	Retrospective cohort	Psychological readiness assessment at 5 years post-surgery can predict RTS practices.
Faleide et al., 2021b [97]	To determine whether psychological readiness scores at 12 months predict RTS at 24 months.	Prospective cohort	Psychological readiness assessment at 9 months post-surgery can predict RTS practices.
Fältström et al., 2016 [98]	To investigate whether player-related factors or the characteristics of the ACL injury were associated with the return to playing football in females after ACLR.	Cross-sectional	Psychological readiness assessment at 18 months post-surgery can predict RTS practices.
Hurley et al., 2022b [99]	To analyze factors associated with non-RTS following open Latarjet.	Retrospective cohort	Psychological readiness assessment at 40 months post-surgery can predict RTS practices.
Hurley et al., 2022c [100]	To analyze factors associated with non-RTS following ABR.	Retrospective cohort	Psychological readiness assessment at two years post-surgery can predict RTS practices.
Langford et al., 2009 [101]	To determine the relationship between psychological readiness and athletic performance levels upon RTS.	Prospective cohort	Psychological readiness at 6 months post-ACLR surgery can predict RTS practices.
Li et al., 2023 [102]	To evaluate the rate of return to sport following bilateral the Medial Patellofemoral Ligament reconstruction compared to a unilateral comparison group.	Retrospective cohort	Psychological readiness assessment can predict RTS practices.
Liew et al., 2022 [103]	To quantify the association between factors associated with return to sport using network analysis.	Cross-sectional	Psychological readiness assessment at 12 months can predict RTS practices.
Manara et al., 2022 [104]	To determine factors associated with repeat ACL injury and return to soccer.	Retrospective case–control	Psychological readiness assessment at 5 years post-ACLR can predict RTS practices.
Müller et al., 2015 [105]	To find predictive parameters for a successful resumption of the pre-injury level of sport.	Prospective cohort	Psychological readiness assessment at 6 months post-ACLR can predict RTS practices.
Slater et al., 2023 [106]	To examine the relationship between biopsychosocial factors and athletic performance levels at 12 months post-RTS.	Prospective single-cohort	Psychological readiness assessments conducted at 3-, 6-, and 12-month post-ACL injury can predict RTS practices.
Toale et al., 2021 [107]	To evaluate the reasons why athletes do not RTS following ACLR.	Prospective cohort	Psychological readiness assessment at 2 years post-ACLR can predict RTS practices.
van Haren et al., 2023 [108]	To develop models to predict RTS and performance in individuals after ACLR.	Prospective cohort	Psychological readiness assessment, one year after discharge from rehabilitation, can predict RTS practices.
Webster and Feller, 2022 [109]	To determine whether psychological readiness scores at 6 months can predict return to competitive sport at 12 months.	Prospective cohort	Psychological readiness assessment at 6 months post-ACLR surgery can predict RTS practices at 12 months.
Webster et al., 2022 [110]	To report determinants of RTS after ACLR in male athletes.	Prospective cohort	Psychological readiness assessment at 12 months post-ACLR can predict RTS practices.
**(3) Predicting Quality of Life Related to Injury and Reinjury Rates**			
Almeida et al., 2023 [111]	To investigate the combinations of variables that comprise the biopsychosocial model domains to identify clinical profiles of risk and protection of second ACL injury.	Prospective cohort	Psychological readiness assessment at 1-year post-surgery can predict the rate of reinjury.
Azevedo Tavares et al., 2023 [112]	To investigate the relationship between psychological readiness and quality of life after ACLR.	Cross-sectional	Psychological readiness assessment can predict knee-related quality of life.
McPherson et al., 2019a [113]	To determine whether psychological readiness to RTS is associated with second ACL injury.	Prospective cohort	Psychological readiness assessment at 12 months post-surgery can predict the rate of reinjury.
Olds and Webster, 2021 [114]	To examine how the SI-RSI is associated with the Western Ontario Shoulder Instability Index.	Cross-sectional	Psychological readiness assessment at 9 months post-shoulder injury can predict the shoulder-related quality of life.
Pasqualini et al., 2024 [115]	To evaluate the predictive ability of psychological readiness to RTS on clinical outcomes and recurrences in athletes who undergo RTS following shoulder instability surgery.	Retrospective cohort	Psychological readiness assessed at 6 months post-shoulder instability surgery can predict the rate of reinjury within two years post-surgery.
Sonesson et al., 2021 [116]	To describe a consecutive cohort of people with a non-surgically treated ACL injury and evaluate correlations between functional performance and patient-reported outcome measures.	Cross-sectional	Psychological readiness assessed two years after an ACL injury can predict both the quality of life at the injury site.
Ueda et al., 2024 [117]	To investigate whether psychological readiness in the early postoperative period can predict the occurrence of a second ACL injury within 24 months after primary ACLR.	Prospective cohort	Psychological readiness assessment at 3 months post-ACLR can predict the reinjury rate within 24 months after ACLR.
**(4) Predicting Post-Return Sport Performance Levels and Physical Activity Levels**			
Albano et al., 2020 [41]	To develop a clinical decision algorithm that could predict RTS after ACLR.	Cross-sectional	Psychological readiness assessment at 3 years post-surgery can predict the level of sports performance after RTS.
Ardern et al., 2013 [118]	To determine whether psychological factors predicted return to the preinjury level of sport by 12 months after ACLR surgery.	Prospective case–control	Psychological readiness at 4 months post-surgery can predict the level of sports performance after RTS.
Beischer et al., 2023 [119]	To characterise patients who had returned to their pre-injury PA or higher at 18 months and maintained that level of PA 3–5 years after ACLR.	Prospective cohort	Psychological readiness assessment at 3- to 5-year post-surgery can predict pre-injury physical activity levels after RTS.
Faleide et al., 2021a [25]	To examine the predictive ability of psychological readiness assessment on return to the preinjury level of sport and reinjury.	Prospective cohort	Psychological readiness assessment at 9 months post-surgery can predict returning to preinjury sport in 2 years.
Garra et al., 2024 [120]	To compare clinical outcomes, rate of RTS, and psychological readiness among patients undergoing ACLR with and without concomitant Segond fracture.	Retrospective cohort	Psychological readiness assessment at 6 years post-surgery can predict sport activity participation.
Hopper et al., 2024 [121]	To examine factors correlated with psychological readiness to return to activity after ACLR.	Cross-sectional study	Psychological readiness assessment at 8 months post-surgery can predict post-return activity levels.
Kitaguchi et al., 2020 [122]	To identify independent predictive factors for RTS.	Prospective cohort	Psychological readiness assessment at 6 months post-ACLR surgery can predict the level of sports activity at 1-year post-surgery.
Rossi et al., 2022 [123]	To evaluate the predictive ability of the SI-RSI for RTS outcomes.	Prospective cohort	Psychological readiness assessment at 12 months post-surgery can predict the pre-injury sport level after RTS.
Sadeqi et al., 2018 [124]	To analyze the progression of the ACL-RSI score from preoperatively to 2-year follow-up.	Prospective cohort	Psychological readiness assessment at 2 years post-ACLR can predict the pre-injury sport level after RTS.
Slagers et al., 2021a [125]	To determine the relationship between psychological readiness and tendinopathy severity, sport participation, and satisfaction with activity level and tendon function.	Cross-sectional	Psychological readiness can predict returning to the pre-injury level of sports.
Slagers et al., 2021b [126]	To explore the association between psychological factors during rehabilitation and functional outcome 12 months after ATR.	Prospective cohort	Psychological readiness assessment at 6 and 12 months after ATR can predict post-injury sport level after RTS.
Stigert et al., 2023 [127]	To investigate whether patient-reported outcomes are associated with physical inactivity after ACLR.	Retrospective case–control	Psychological readiness assessment at 18 months after ACLR can predict levels of physical activity 5–8 years post-surgery.
Suzuki et al., 2023 [128]	To examine the association between psychological readiness to RTS and subjective RTS level at 12 months after ACLR.	Prospective case–control	Psychological readiness assessment at 6 and 12 months post-ACLR can predict post-injury sport level after RTS.

If not otherwise specified, the age of the participants is presented as mean ± SD; *IQR* interquartile range, *N/A* No data or not applicable, *BMI* Body Mass Index, *RTS* Return to Sport, *ACLR* Anterior Cruciate Ligament Reconstruction, *ACL-RSI* Anterior Cruciate Ligament-Return to Sport after Injury, *IKDC* International Knee Documentation Committee, *KOOS* Knee Injury and Osteoarthritis Outcome Score, *BEAR* Bridge-enhanced ACL restoration, *I-PRRS* The Injury Psychological Readiness to Return to Sport, *SI-RSI* Shoulder Instability-Return to Sport after Injury, *CSAS* Cincinnati Sports Activity Scale, *ABR* arthroscopic Bankart repair, *MPFL-RSI* medial patellofemoral ligament-return to sport after injury, *PA* physical activity, *ATR* Achilles tendon rupture, *SLAP* superior-labrum anterior–posterior

^*^ The Tegner Activity Scale is a tool used to assess an individual’s activity level before and after sports injuries or surgeries (Briggs et al., 2009)

^†^ The IKDC (International Knee Documentation Committee) classification system is primarily used to assess knee function and activity level following an injury (Higgins et al., 2007)

^‡^ The Marx Activity Score is a tool used to assess an individual’s activity level and function following a knee injury or surgery (Marx et al., 2001)

^#^The Cincinnati Sports Activity Scale is used to assess the level of sports activity of a patient prior to the injury (Barber-Westin & Noyes, 1999)

**Table 2:** Summary of Participants, Injury, and Measurement Information from the Included Articles (Alphabetical Order by Domain)

**Participants Information**		**InjuryInformation**												**Measurement Information**
**Author(s) and Year**	**Population Groups**	**Types ofSport**	**Sports Experience**	**Number of Participants and Gender (males/females)**	**Age (Year)**	**Anthropometric Measurements**	**Treatment Methods**	**Time Duration from Injury to Surgery**	**Type of Surgery**	**Graft Type**	**Surgical History**	**Injury Context**	**Injury Mechanism**	**Measurement Time, Tools, and Score**
**(1) Predicting Physical Recovery Outcomes**														
Aizawa et al., 2020 [70]	N/A	Basketball (n = 7) Soccer (n = 8) Futsal (n = 2) Volleyball (n = 6) Badminton (n = 5) Tennis (n = 1) Frisbee (n = 1)	Preinjury Tegner score*: median (IQR) = 7.5 (2.0) Preinjury sports participation time: median (IQR) = 4.0 (5.0) hours per week	N = 30 (8/22)	Median (IQR) = 20.0 (7.3)	Height = 164.4 ± 0.1 (cm) Weight = 58.7 ± 8.2 (kg) BMI = 21.7 ± 2.0 (kg/m²)	Surgical treatment	Median (IQR) = 66.5 (97.3) days	ACLR	Hamstring tendon autograft (N = 30)	Primary	N/A	N/A	187.5 ± 15.9 days postoperatively Japanese version of the ACL-RSI score = 65.1 ± 18.3
Aizawa et al., 2022 [71]	N/A	Collision sports (n = 6) Contact (n = 33) Limited contact (n = 7) Non-contact (n = 8)	Preinjury Tegner score: median (IQR) = 8.0 (2.0) Preinjury sports participation time: median (IQR) = 6.0 (8.0) hours per week	N = 54 (21/33)	Median (IQR) = 20.0 (4.3)	Height = 165.8 ± 8.3 (cm) Weight: Median (IQR) = 61.0 (16.5) (kg) BMI: Median (IQR) = 21.9 (3.0) (kg/m²)	Surgical treatment	Median (IQR) = 66.0 (76.5) days	ACLR	Semitendinosus (n = 44) Gracilis tendon in addition to semitendinosus (n = 4) Patellar tendon autograft (n = 6)	Primary	N/A	Non-contact injury (n = 34) Indirect contact injury (n = 14) Direct contact injury (n = 6)	Median (IRQ) = 186.5 (15.3) days postoperatively Japanese version of the ACL-RSI score = 64.8 ± 18.1
Baez et al., 2023 [72]	N/A	N/A	N/A	N = 101 (43/58)	18.5 ± 2.7	24.6 ± 4.8 (kg/m²)	Surgical treatment	N/A	ACLR	Bone-tendon-bone (n = 21) Hamstring (n = 74) Allograft (n = 5)	Primary	N/A	N/A	8.0 ± 1.7 months postoperatively Persistent knee symptoms present group: ACL-RSI score = 59.5 ± 19.5 Persistent knee symptoms absent group: ACL-RSI score = 76.4 ± 22.0
Borawski et al., 2024 [73]	N/A	N/A	N/A	N = 37 (20/17)	20.6 ± 1.8	Height = 1.76 ± 0.1 (m) Weight = 81.5 ± 15.3 (kg)	Surgical treatment	N/A	ACLR	Bone tendon bone patellar (n = 23) Quadriceps tendon (n = 12) Hamstring (n =2)	N/A	N/A	N/A	18.3 ± 8.9 months postoperatively ACL-RSI score = 59.4 ± 22.4
Correa et al., 2023 [74]	Athlete	Football (N = 35)	Had at least 100 hours of soccer practice per year Competitive level before injury	N = 35 (35/0)	Ready RTS group (ACL-RSI score ≥ 60): 23.00 ± 5.89 Not-ready RTS group (ACL-RSI score **<** 60): 21.00 ± 3.78	Ready RTS group: Height = 177.55 ± 6.71 cm Weight = 75.44 ± 9.37 kg Not-ready RTS group: Height = 178.87 ± 5.04 cm Weight = 74.30 ± 7.87 kg	Surgical treatment	Ready RTS group: 17.50 ± 4.29 days Not-ready RTS group: 18.33 ± 4.40 days	ACLR	Hamstring tendon autograft (N = 35)	Primary	N/A	N/A	Ready RTS group: 7.40 ± 1.43 months postoperatively Brazilian version of the ACL-RSI score = 66.5 ± 5.2 Not-ready RTS group: 7.60 ± 1.50 months postoperatively Brazilian version of the ACL-RSI score = 46.1 ± 10.5
Cronstrom et al., 2023b [75]	N/A	N/A	Preinjury Tegner score: median (range) = 7 (5–9)	N = 143 (71/72)	25.0 ± 5.7	BMI = 24.3 ± 3.6 (kg/m²)	Surgical treatment	Median (range) = 9 (4–21) months	ACLR	Hamstring (n = 113) Patella (n = 17) Donor (graft type unknown) (n = 1) Not reported (n = 12)	Primary	N/A	Contact injury (n = 47) Non-contact injury (n = 80) Other injury (such as trauma) (n = 3) Not reported (n = 13)	12.5 ± 2.0 months postoperatively Male: ACL-RSI score = 47.3 ± 24.5 Female: ACL-RSI score = 44.2 ± 24.9
Dombrowski et al., 2024 [76]	N/A	N/A	N/A	N = 127 (63/64)	Men: 20.14 ± 6.90 Women: 19.13 ± 8.51	Men: 85.19 ± 18.28 (kg) Women: 67.61 ± 12.91 (kg)	Surgical treatment	N/A	ACLR	Allograft (n = 12) Autograft (n = 114) Unknown (n = 1)	N/A	N/A	N/A	6-8 months postoperatively Men: ACL-RSI score = 76.40 ± 18.76 Women: ACL-RSI score = 75.90 ± 19.26
Erickson et al., 2022 [77]	Athlete	N/A	Preinjury Tegner score ≥ 5 Recreational level Competitive level	N = 30 (17/13)	18.3 ± 4.4	BMI = 23.8 ± 2.6 (kg/m²)	Surgical treatment	≤ 4 months	ACLR	Patellar tendon (n = 23) Hamstring (n = 7)	Primary	N/A	N/A	105.1 ± 10.3 days postoperatively ACL-RSI score = 58.4 ± 23.2
Fones et al., 2020 [78]	Athlete	Soccer (n = 22) Basketball (n = 9) Football (n = 10) Softball/baseball (n = 9) Lacrosse (n = 9) Gymnastics/cheerleading (n = 6) Other (n = 9)	Recreational level (n = 10) Competitive level (n = 64)	N = 74 (28/46)	Tatol: 15.9 ± 1.5 RTS group: 15.8 ± 1.4 Non-RTS group: 16.0 ± 1.6	Tatol: BMI = 24.3 ± 5.3 (kg/m²) RTS group: BMI = 23.8 ± 4.2 (kg/m²) Non-RTS group: BMI = 25.6 ± 7.3 (kg/m²)	Surgical treatment	RTS group: 51.2 ± 33.3 days Non-RTS group: 91 ± 84.9 days	ACLR	Bone-patellar tendon-bone autograft (n = 8) Hamstring autograft (n = 65) Hamstring allograft (n = 1)	Primary	N/A	N/A	4.0 ± 2.0 years postoperatively RTS group: ACL-RSI score = 81.6 ± 20.4 Non-RTS group: ACL-RSI score = 52.7 ± 26.7
Hart et al., 2020 [79]	Athlete and non-athlete	N/A	Competitive level (n = 91)	N = 118 (76/42)	31 ± 9	BMI = 26 ± 4 (kg/m²)	Surgical treatment	N/A	ACLR	Hamstring-tendon autograft (N = 118)	Primary	N/A	N/A	12.7 ± 0.1 months postoperatively ACL-RSI score = 53 ± 20
Högberg et al., 2023 [80]	N/A	N/A	Preinjury Tegner score: median (range) = 8 (6 –10)	N = 137 (73/64)	24.8 ± 8.4	Height = 174.8 ± 9.2 (cm) Weight = 74.2 ± 11.9 (kg) BMI = 24.2 ± 2.6 (kg/m²)	Surgical treatment	245.5 ± 429.0 days	ACLR	Hamstring tendon autograft (N = 137)	N/A	N/A	N/A	8 months postoperatively ACL-RSI score = 74.8 ± 19.0 12 months postoperatively ACL-RSI score = 79.4 ± 24.4
Hurley et al., 2022a [81]	Athlete	Collision sports (n = 102) Non-collision sport (n = 42)	Professional level (n = 9) Competitive level (n = 95) Recreational level (n = 40)	N = 144 (132/12)	26.9 ± 8.1 Median (range) = 25 (17–40)	N/A	Surgical treatment	N/A	ABR	N/A	N/A	N/A	N/A	75.7 ± 13.6 months postoperatively SI-RSI score = 63.7 ± 25.7
Legnani et al., 2023 [82]	Athlete	Contact sports (e.g., soccer, basketball, and rugby) (n = 21) Non-contact sports (e.g., volleyball, skiing, cycling, running, swimming, and tennis) (n = 28)	Competitive level: Agonistic level (n = 11) Amateurs level (n = 20) Preinjury Tegner score: 4.2 ± 2.4	N = 31 (29/2)	Age at ACLR: 34.2 ± 11.3	BMI = 25.4 ± 3.7 (kg/m²)	Surgical treatment	2.7 ± 1.1 months	ACLR	Doubled autologous hamstring graft	Primary	N/A	Contact injury (n = 21) Non-contact injury (n = 28)	Preoperatively Italian version of the ACL-RSI score = 51.9 ± 13.0 6 months postoperatively Italian version of the ACL-RSI score = 77.1 ± 14.6
Milewski et al., 2023 [83]	N/A	Soccer (n = 50) Basketball (n = 32) Lacrosse (n = 19) Football (n = 13) Baseball/softball (n = 7) Cheerleading (n = 4) Dance (n = 3) Gymnastics (n = 3) Hockey (n = 6) Field hockey (n = 5) Running (n = 4) Track (n = 5) Skiing (n = 7) Marital arts (n = 3) Other (n = 15)	N/A	N = 176 (69/107)	17.1 ± 3.1	BMI = 24.1 ± 4.6 (kg/m²)	Surgical treatment	N/A	ACLR	Hamstring autograft (n = 128) Bone-patellar tendon-bone autograft (n =31) Iliotibial band autograft (n =13) Quadriceps autograft (n =4)	Primary	In sports (n = 165) Out of sports (n = 11)	N/A	6 months postoperatively ACL-RSI score = 61.5 ± 21.2
Nagelli et al., 2019 [84]	Athlete	N/A	Recreational level	N = 18 (9/9)	20 ± 7.4	Height = 170 ± 7.05 (cm) Weight = 72.7 ± 14.0 (kg)	Surgical treatment	N/A	ACLR	Hamstring autograft (N = 18)	N/A	N/A	N/A	8.5 ± 4.2 months postoperatively ACL-RSI score = 66.7 ± 22.5
Peebles et al., 2021 [85]	N/A	N/A	N/A	N = 38 (22/16)	16.3 ± 1.9	Height = 174.4 ± 10.5 (cm) Weight = 75.0 ± 16.6 (kg)	Surgical treatment	N/A	ACLR	Bone-patellar tendon-bone autograft (n = 19) Hamstring autograft (n = 19)	Primary	In sports (n = 37) Out of sports (n = 1)	N/A	25.7 ± 6.2 weeks postoperatively ACL-RSI score = 74.4 ± 25.6
Sanborn et al., 2022 [86]	N/A	N/A	Preinjury IKDC level^†^: Level I (n =75) Level II/III (n = 25) Preinjury Marx activity score^‡^: BEAR group: median (IQR) = 16 (13-16) ACLR group: median (IQR) = 16 (13-16)	N = 100 (44/56)	BEAR group: median (IQR) = 17 (16–20) ACLR group: median (IQR) = 17 (15–23)	BEAR group: BMI = 24.7 ± 3.8 (kg/m²) ACLR group: BMI = 23.3 ± 4.5 (kg/m²)	Surgical treatment	BEAR group: median (IQR) = 36 (29–42) day ACLR group: median (IQR) = 39 (33–43) day	BEAR (n = 65) ACLR (n =35)	Quadrupled hamstring (n = 33) Bone–patellar tendon–bone (n = 2)	Primary	In sports (n = 99) Out of sports (n = 1)	Contact injury (n = 23) Non-contact injury (n = 77)	6 months postoperatively BEAR group: ACL-RSI score = 71.1 ± 2.9 ACLR group: ACL-RSI score = 58.2 ± 3.9 12 months postoperatively BEAR group: ACL-RSI score = 69.7 ± 2.9 ACLR group: ACL-RSI score = 64.8 ± 3.9 24 months postoperatively BEAR group: ACL-RSI score = 68.8 ± 2.9 ACLR group: ACL-RSI score = 71.2 ± 3.9
Schilaty et al., 2023 [87]	Athlete	N/A	Competitive level (n = 13) Recreational level (n = 5) Sedentary level (n = 1)	N = 19	Low ACL-RSI score group ( ACL-RSI score < 61): n = 9 20.0 ± 3.3 High ACL-RSI score group ( ACL-RSI score ≥ 61): n = 10 18.1±2.8	Low ACL-RSI score group: Height = 175.5 ± 5.5 (cm) Weight = 84.9 ± 9.6 (kg) High ACL-RSI score group: Height = 172.0 ± 11.4 (cm) Weight = 71.2 ± 18.0 (kg)	Surgical treatment	Median (IQR) = 37 (25-94) days	ACLR	Autograft bone–tendon–bone (n = 9) Autograft hamstring (n = 5) Allograft (n = 1) None (n = 24)	Primary (n = 12) Revision ACLR (n = 7)	N/A	Contact injury (n = 5) Non-contact injury (n = 14)	Median (IQR) = 37 (25–94) days postoperatively ACL-RSI score < 61 (n = 9) ACL-RSI score ≥ 61 (n = 10)
Sugarman et al., 2022 [88]	Athlete	N/A	Current Tegner score = 7.28 ± 1.44	18 (10/8)	20.2 ± 6.35	Height = 173.15 ± 10.06 (cm) Weight = 76.64 ± 18.32 (kg)	Surgical treatment	N/A	ACLR	N/A	Primary	N/A	N/A	High ACL-RSI score group: 8.74 ± 1.54 months postoperatively ACL-RSI score = 83.06 ± 6.22 Low ACL-RSI score group: 9.50 ± 2.75 months postoperatively ACL-RSI score = 61.76 ± 8.00
Ueda et al., 2022 [89]	N/A	N/A	Preinjury Tegner score = 7.6 ± 1.4	144 (82/62)	25.8 ± 11.9	BMI = 22.6 ± 3.0 (kg/m²)	Surgical treatment	25.8 ± 11.9 months	ACLR	Bone–patellar tendon–bone graft (n = 6) Hamstring tendon autograft (n = 138)	Primary	N/A	N/A	12 months postoperative Japanese version of the ACL-RSI score = 70.3 ± 21.0
Webster and Feller, 2020 [90]	N/A	N/A	Preinjury IKDC level: Level I (n = 381) Level II/III (n = 69) Participated in sport on a weekly basis before injury	450 (274/176)	24 ± 7	N/A	Surgical treatment	N/A	ACLR	Hamstring tendon (semitendinosus and gracilis) autograft (n = 391) Patellar tendon autograft (n = 12) Quadriceps tendon autograft (n = 47)	Primary	N/A	N/A	6.5 ± 0.6 months postoperative ACL-RSI score ≥ 65 (n = 146) ACL-RSI score < 65 (n = 304)
Webster et al., 2018 [91]	N/A	N/A	Preinjury sport frequency 4–7 day per week (n = 285) 1–3 day per week (n = 350)	N = 635 (389/246)	28 ± 10	N/A	Surgical treatment	11.8 ± 35 months	ACLR	Hamstring tendon autograft (N = 635)	Primary	N/A	N/A	12 ± 1 months postoperatively ACL-RSI score = 65 ± 23
Zarzycki et al., 2018 [92]	Athlete	N/A	Regularly participated in cutting, pivoting, and jumping sports (> 50 hours per year)	79 (40/39)	Low ACL-RSI group (ACL-RSI score ≤ 47; n =19): 22.3 ± 6.5 Middle ACL-RSI group (ACL-RSI score ≥ 48 and ≤ 78; n = 40): 20.7 ± 7.7 High ACL-RSI group (ACL-RSI score ≥ 79; n = 20): 21.0 ± 8.7	Low ACL-RSI group: BMI = 25.9 ± 4.0 (kg/m²) Middle ACL-RSI group: BMI = 26.3 ± 3.3 (kg/m²) High ACL-RSI group: BMI = 26.3 ± 3.3 (kg/m²)	Surgical treatment	N/A	ACLR	N/A	Primary	N/A	N/A	Low ACL-RSI group: 24.1 ± 8.8 weeks postoperative ACL-RSI score = 24.1 ± 8.8 Middle ACL-RSI group: 23.3 ± 7.1 weeks postoperative ACL-RSI score = 23.3 ± 7.1 High ACL-RSI group: 23.3 ± 9.2 weeks postoperative ACL-RSI score = 23.3 ± 9.2
Zwolski et al., 2023 [93]	Athlete	N/A	Preinjury Tegner score: Median (range) = 9 (7 – 10)	41 (12/29)	Median (IQR) = 16.6 (16.0 – 18.3)	Height = 168.8 ± 9.5 (cm) Weight = 70.7 ± 17.5 (kg)	Surgical treatment	N/A	ACLR	Hamstring autograft (n = 23) Patellar tendon autograft (n = 9) Quadriceps tendon autograft (n = 9)	Primary	N/A	N/A	3.7 ± 2.4 weeks after RTS ACL-RSI score: median (range) = 88.3 (33.3–100)
**(2) Predicting Return to Sport Practices**														
Aizawa et al., 2024 [94]	N/A	Collision (n = 6) Contact (n = 38) Limited contact (n = 7) Non-contact (n = 8)	Preinjury Tegner score: median (IQR) = 8.0 (2.0) Preinjury sports participation time: median (IQR) = 6.0 (8.0) hours per week	N = 59 (24/35)	Median (IQR) = 20.0 (6.0)	Height: = 166.3 ± 8.6 (cm) Weight: median (IQR) = 61.0 (19.0) (kg) BMI = 22.9 ± 2.9 (kg/m²)	Surgical treatment	Median (IQR) = 67.0 (70.0) days	ACLR	Semitendinosus (n = 48) Gracilis tendon in addition to semitendinosus (n = 4) Patellar tendon autograft (n = 7)	Primary	N/A	Non-contact injury (n = 37) Indirect contact injury (n = 15) Direct contact injury (n = 7)	Median (IQR) = 187.0 (14.0) days postoperatively ACL-RSI score = 63.8 ± 17.9
Beischer et al., 2019 [95]	Athlete	N/A	8 months postoperatively Tegner score: 6 (n = 23) 7 (n = 61) 8 (n = 98) 9 (n = 133) 10 (n = 69) 12 months postoperatively Tegner score: 6 (n = 19) 7 (n = 38) 8 (n = 84) 9 (n = 93) 10 (n = 37)	8 months postoperatively: N = 384 (192/192) 12 months postoperatively: N = 271 (129/142)	8 months postoperatively: 22.1 ± 4.5 12 months postoperatively: 22.2 ± 4.6	8 months postoperatively Height = 175 ± 9.7 (cm) Weight = 73 ± 13 (kg) BMI = 24 ± 3 (kg/m²) 12 months postoperatively Height = 175 ± 9.6 (cm) Weight = 72 ± 11.9 (kg) BMI = 24 ± 3 (kg/m²)	Surgical treatment	8 months postoperatively 8.7 ± 13.7 months 12 months postoperatively 7.7 ± 12.6 months	ACLR	N/A	Primary Revision ACLR	N/A	N/A	8 months postoperatively 15-20 years group: Swedish version of the ACL-RSI score: median (range) = 58.3 (17.5 – 99.2) 21-30 years group: Swedish version of the ACL-RSI score: median (range) = 55.0 (10.0 – 99.2) 12 months postoperatively 15-20 years group: Swedish version of the ACL-RSI score: median (range) = 63.8 (13.3 – 100.0) 21-30 years group: Swedish version of the ACL-RSI score: median (range) = 58.3 (10.0 – 100.0)
Bohu et al., 2021 [96]	N/A	Overhead sports such as handball, rugby, basketball, judo, tennis, ski, badminton, and volley (n = 173) Non overhead sports such as football, jogging, and cycling (n = 44)	N/A	N = 217 (184/33)	26.8 ± 7.3	BMI = 23.8 ± 3.0 (kg/m²)	Surgical treatment	4.4 ± 4.9 year	Arthroscopic Latarjet procedure (n = 23) Mini-open Latarjet procedure (n = 196)	N/A	Primary	In sports (n = 182) Out of sports (n = 35)	Contact injury (n = 152) Non-contact injury (n = 65)	Preoperative SI-RSI score = 51 ± 26 Postoperative at 8 months SI-RSI score = 70 ± 23
Colasanti et al., 2023 [33	Athlete	Golf (n = 13) Weightlifting/Powerlifting/CrossFit (n = 29) Swimming (n = 20) Baseball (n = 39) Rock climbing (n = 10) Tennis (n = 21) Mixed martial arts (n = 16) Basketball (n = 38) Volleyball (n = 7) Football/rugby (n = 12) Hockey (n = 5)	Recreational level (n = 127) High school level (n = 6) College level (n = 59) Semiprofessional level (n = 11) Professional level (n = 6)	N = 209 (178/31)	RTS group: 34.2 ± 8.4 Non-RTS group: 34.3 ± 8.9	N/A	Surgical treatment	N/A	SLAP repair (n = 106) Biceps tenodesis (n = 103)	N/A	N/A	N/A	N/A	RTS group: 60.7 ± 24.9 months postoperatively SLAP-RSI score = 76.8 ± 21.3 Non-RTS group: 57.8 ± 28.4 months postoperatively SLAP-RSI score = 50.0 ± 28.0
Faleide et al., 2021b [97]	Athlete	Soccer (n = 64) Handball (n = 13) Alpine skiing (n = 12)	Competitive level: Elite level (n = 7) Medium/high competitive level (n = 38) Low competitive level (n = 43) Recreational level (n = 44)	N = 132 (75/57)	28.7 ± 9.8	N/A	Surgical treatment	N/A	ACLR	Bone-patellar tendon-bone autograft (n = 85) Hamstring tendon autograft (n = 47)	Primary (n = 123) Revision ACLR (n = 9)	N/A	N/A	9 months postoperatively ACL-RSI score = 55.5 ± 23.7
Fältström et al., 2016 [98]	Athlete	Football (N = 182)	Elite level (n = 12) Sub-elite level (n = 56) Recreational level (n = 109)	N = 182 (0/182)	RTS group: 18.5 ± 2.3 Not-RTS group: 19.1 ± 3.0	Current players group: BMI = 22.3 ± 2.3 (kg/m²) Had not returned group: BMI = 22.3 ± 2.7 (kg/m²)	Surgical treatment	0–90 days (n = 47) 91–365 days (n = 102) > 365 days (n = 20)	ACLR	Hamstring (n = 177) Patellar tendon (n = 2) Others (n = 3)	Primary	N/A	Contact injury (n = 71) Non-contact injury (n = 101)	Current players group: postoperatively time: median (IQR) = 17.5 (13.7) months Swedish version of the ACL-RSI score = 6.6 ± 1.9 Had not returned group: postoperatively time: median (IQR) = 18.3 (12.3) months Swedish version of the ACL-RSI score = 3.9 ± 2.0
Hurley et al., 2022b [99]	N/A	Collision sports (n = 78) Non-collision sports (n = 27)	N/A	N = 105 (105/0)	Total sample: 26.8 RTS group: 26.2 ± 4.9 Non-RTS group: 27.9 ± 8.3	N/A	Surgical treatment	N/A	Open Latarjet procedure	N/A	Primary (n = 75) Non- primary (n = 30)	N/A	N/A	RTS group: 39.3 ± 24.9 months postoperatively SI-RSI score = 74.5 ± 19.8 Non-RTS group: 41.5 ± 25.3 months postoperatively SI-RSI score = 41.5 ± 21.9
Hurley et al., 2022c [100]	N/A	Collision sports (n = 100) Non-collision sports (n = 108)	N/A	N = 208 (184/24)	RTS group: 28.3 ± 8 Non-RTS group: 29.4 ± 9	N/A	Surgical treatment	N/A	ABR	N/A	Primary (n = 109) Non- primary (n = 198)	N/A	N/A	RTS group: 62.8 ± 22.6 months postoperatively SI-RSI score = 68.9 ± 22.0 Non-RTS group: 62.4 ± 23 months postoperatively SI-RSI score = 39.8 ± 24.6
Langford et al., 2009 [101]	Athlete	N/A	CSAS^#^ Level 1 Level 2	N = 87 (55/32)	27.48 ± 5.72	N/A	Surgical treatment	18 weeks (range: 2 – 216 weeks)	ACLR	Bone-patellar tendon-bone (n = 4) Hamstring tendon (n = 83)	Primary (n = 82) Non- primary (n = 5)	N/A	N/A	3 months postoperatively ACL-RSI score = 55.73 ± 16.87 6 months postoperatively ACL-RSI score = 57.56 ± 17.83 12 months postoperatively ACL-RSI score = 65.40 ± 18.50
Li et al., 2023 [102]	N/A	N/A	Bilateral group: Preinjury Tegner score: median (range) = 6 (2–10) Unilateral group: Preinjury Tegner score: median (range) = 6 (2–10)	N = 63 (30/33)	Bilateral group: 24.6 ± 7.6 Unilateral group: 22.4 ± 6.0	Bilateral group: BMI = 27.2 ± 5.6 (kg/m²) Unilateral group: BMI = 26.4 ± 5.9 (kg/m²)	Surgical treatment	N/A	MPFLR	N/A	Primary	N/A	N/A	Bilateral group: 42.2 ± 28.4 months postoperatively MPFL-RSI score = 50.9 ± 27.4 Unilateral group: 46.8 ± 29.5 months postoperatively MPFL-RSI score = 60.6 ± 29.8
Liew et al., 2022 [103]	Athlete	N/A	Professional level (n = 10) High-level competition sport (n =169) Frequent sport (n =232) Sport sometimes (n = 30)	N = 441 (257/184)	Age at ACLR：24.6 ± 7.4	N/A	Surgical treatment	N/A	ACLR	N/A	Primary	N/A	N/A	12 months postoperatively ACL-RSI score Item 1 to 12: 80.78 ± 22.94 67.34 ± 26.02 59.34 ± 31.15 74.15 ± 25.68 67.72 ± 29.73 50.56 ± 34.47 53.88 ± 31.49 74.71 ± 24.23 54.74 ± 30.93 69.57 ± 31.78 74.92 ± 26.09 67.94 ± 27.95
Manara et al., 2022 [104]	N/A	Football (N = 862)	CSAS Male = 82 Female = 84	N = 862 (666/196)	≤ 18 (n = 89) 19 – 25 (n = 234) > 25 (n = 539)	BMI < 25 (kg/m²) n = 187 BMI > 25 (kg/m²) n = 441	Surgical treatment	Acute surgery (n = 47) Subacute surgery (n = 555) Chronic surgery (n = 260)	ACLR	Hamstring tendon autograft (n = 862)	Primary	In sports (N = 862)	N/A	8.3 years postoperatively ACL-RSI score ≥ 60 (n = 368) < 60 (n = 258)
Müller et al., 2015 [105]	N/A	N/A	Preinjury IKDC level: Level I/II (n = 31) Level III (n = 8) Sport level before injury: Level I/II = 8.6 ± 3.7 hour per week Level III = 9.4 ± 4.1 hour per week	N = 39 (21/18)	RTS group: 31.4 ± 10.3 Non-RTS group: 33.0 ± 10.5	RTS group: Height = 176.0 ± 9.8 (cm) Weight = 74.3 ± 14.2 (kg) Non-RTS group: Height = 173.8 ± 9.5 (cm) Weight = 69.0 ± 14.2 (kg)	Surgical treatment	RTS group: 13.3 ± 19.2 (weeks) Non-RTS group: 30.6 ± 39.3 (weeks)	ACLR	Hamstring autograft (N = 39)	N/A	In sports (n = 35) Out of sports (n = 4)	N/A	6 months postoperatively RTS group: German version of the ACL-RSI score =76.8 ± 15.0 Non-RTS group: German version of the ACL-RSI score = 48.7 ± 27.2
Slater et al., 2023 [106]	N/A	Soccer (n = 22) Floorball (n = 13) Running (n = 11) Strength training (n = 11) Aerobics (n = 7) Cycling/spinning (n = 3) Martial arts (n = 3) Walking (n = 3) Handball (n = 2) Ice hockey (n = 2) Basketball (n = 1) Dance (n = 1) Swimming (n = 1) Volleyball (n = 1) Other (n = 7)	Preinjury Tegner score: median (IQR) = 6.5 (5) Preinjury IKDC Level Level I (n = 39) Level II sports (n = 14) Level III sports (n = 35)	88 (44/44)	Median (IQR) = 27 (11.4)	N/A	Conservative treatment	N/A	N/A	N/A	Primary	N/A	N/A	3 months from ACL injury Swedish version of the ACL-RSI score = 4.6 ± 2.0 6 months from ACL injury Swedish version of the ACL-RSI score = 5.1 ± 2.1 12 months from ACL injury Swedish version of the ACL-RSI score = 5.7 ± 2.5
Toale et al., 2021 [107]	N/A	N/A	N/A	1362 (1019/343)	RTS group: 23.6 ± 7.0 Non-RTS group: 27.2 ± 7.5	N/A	Surgical treatment	N/A	ACLR	Bone-patellar tendon-bone autograft (n = 1087) Hamstring tendon autograft (n = 275)	Primary	N/A	Contact to knee (n = 266) Contact (other than to knee) (n = 211) Non-contact injury (n = 885)	Preoperatively RTS group: ACL-RSI score = 49.3 ± 26.3 Non-RTS group: ACL-RSI score = 40.3 ± 26 24 months postoperative RTS group: ACL-RSI score = 78.7 ± 20.2 Non-RTS group: ACL-RSI score = 41.8 ± 25.6
van Haren et al., 2023 [108]	N/A	Football (n = 131) Handball (n = 14) Korfball (n = 14) Hockey (n = 12) Volleyball (n = 7) Other such as softball, skiing, judo, gymnastics, tennis, and squash (n = 30)	Preinjury Tegner score: 6 (n = 20) 7 (n = 69) 8 (n = 23) 9 (n = 91) 10 (n = 5)	208 (136/72)	23.9 ± 6.7	BMI = 23.2 ± 2.7 (kg/m²)	Surgical treatment	N/A	ACLR	Autograft (n = 195) Allograft (n = 13)	Primary (n = 180) Revision ACLR (n = 28)	N/A	N/A	12 months after discharge from rehabilitation ACL-RSI score = 65.3 ± 21
Webster and Feller, 2022 [109]	Athlete	Australian rules football (n = 39) Netball (n = 34) Basketball (n = 17) Soccer (n = 11) Rugby (n = 4) Skiing (n = 3) Hockey (n = 2) Gymnastics (n = 1) Athletics (long jump) (n = 1) Taekwondo (n = 1)	Elite (n = 4) High-level competition (n = 58) Frequent sports (n = 53)	115 (65/50)	16.2 ± 0.9	N/A	Surgical treatment	N/A	ACLR	Hamstring tendon (n = 94) Unknow (n = 21)	Primary	In sports (n = 113) Out of sports (n = 2)	N/A	6 months postoperative ACL-RSI score = 55.3 ± 19.5 12 months postoperative ACL-RSI score = 71.1 ± 20.2
Webster et al., 2022 [110]	Athlete	Australian Rules football (N = 354)	Competitive level: High-level competition (n = 188) Well-trained and frequent sports (n = 166) Preinjury Marx activity score = 14.0 ± 2.2 Preinjury sport frequency: 4-7 days per week (n = 206) 1-3 days per week (n = 148)	354 (354/0)	23.0 ± 4.8	N/A	Surgical treatment	< 3 months (n = 283) 3 – 6 months (n = 36) > 6 months (n = 35)	ACLR	Hamstring tendon autograft (n = 311) Patellar tendon autograft (n = 9) Quadriceps autograft (n = 34)	Primary	In sports (n = 343) Out of sports (n = 11)	Contact injury (n = 143) Non-contact injury (n = 181)	3.1 ± 1.0 year postoperative ACL-RSI score < 50 (n = 86) ACL-RSI score > 50 (n = 85)
**(3) Predicting Quality of Life Related to Injury and Reinjury Rates**														
Almeida et al., 2023 [111]	N/A	Soccer (n = 54) Handball Basketball Volleyball Tennis Wrestling	Preinjury IKDC level: Level I Level II	N = 88 (75/13)	26.9 ± 6.3	Weight = 82.6 ± 14.9 (kg) Height = 1.74 ± 0.07 (m) BMI = 27.3 ± 4.1 (kg/m²)	Surgical treatment	N/A	ACLR	Hamstring tendon autograft (n = 77) Patellar (n = 11)	N/A	N/A	N/A	11.5 ± 5.8 months postoperatively Brazilian Portuguese version of the ACL-RSI score = 45.8 ± 16.9
Azevedo Tavares et al., 2023 [112]	Athlete	N/A	Preinjury Tegner score = 7.7 ± 1.5 Recreational level	N = 131 (115/16)	29.9 ± 7.7	Height = 173.5 ± 7.9 (cm) Weight = 78.6 ± 12.4 (kg) BMI = 26.3 ± 3.2 (kg/m^2^)	Surgical treatment	N/A	ACLR	Hamstring (n = 96) Patellar (n = 30) Quadriceps (n = 4) Achilles tendon (allograft) (n = 1)	Primary	N/A	Contact injury (n = 27) Non-contact injury (n = 104)	Postoperative time: 0.5-2 years (n = 82) 2-5 years (n = 23) > 5 years (n = 26) ACL-RSI score = 56.1 ± 18.4
McPherson et al., 2019a [113]	N/A	N/A	N/A	N = 329 (211/118)	Age at ACLR：25.3 ± 8.7	Height = 176.5 ± 9.6 (cm) Weight = 77.0 ± 13.7 (kg)	Surgical treatment	N/A	ACLR	Hamstring autograft (n = 312) Patellar tendon autograft (n = 10) Other graft type (i.e., synthetic graft, and quadriceps tendon autograft) (n = 7)	Primary	N/A	N/A	Preoperative ACL-RSI score = 49.5 ± 21.8 12 months postoperatively ACL-RSI score = 66.4 ± 22.4
Olds and Webster, 2021 [114]	Athlete	Rugby (n = 38) Touch rugby (n = 8) Field hockey (n = 7) Netball (n = 6) Weightlifting (n = 3) Soccer (n = 3) Kayaking (n = 3) Surfing (n = 2) Swimming (n = 2) Tennis/badminton (n = 2) Boxing (n = 2) Rock climbing (n = 2) Javelin (n = 1) Karate (n = 1)	Participated in sports on a weekly basis before dislocation	N = 80 (62/18)	22 (range: 15–45)	N/A	Surgical treatment (n = 27) Conservative treatment (n = 53)	N/A	Shoulder surgery for dislocation	N/A	N/A	N/A	N/A	9 months from a shoulder dislocation SI-RSI score = 47.06 ± 15.9
Pasqualini et al., 2024 [115]	Athlete	Contact sports (n = 88) Non-contact sports (n = 71)	Competitive level (n = 96) Recreational level (n = 53)	N = 159 (136/23)	Ready RTS group (SI-RSI score ≥ 55) 26.7 ± 0.3 Not ready RTS group (SI-RSI score < 55) 25.9 ± 7	N/A	Surgical treatment	N/A	Latarjet procedure (n = 69) ABR (n = 80)	N/A	Primary	N/A	Contact injury (n = 88) Non-contact injury (n = 71)	6 months postoperatively Ready RTS group: SI-RSI score = 78.7 ± 1.2 Not ready RTS group: SI-RSI score = 39.2 ± 1.5
Sonesson et al., 2021 [116]	Athlete (n = 60) Non-athlete (n = 8)	Football (n = 31) Motocross (n = 6) Floorball (n = 3) Horse riding (n = 3) Other (n = 17)	Preinjury Tegner score: median (range) = 8 (7–9) Sport level Elite level (n = 4) Competitive level (n = 39) Recreational level (n = 17) Non-athlete (n = 8)	68 (38/30)	30.6 ± 8.3	N/A	Conservative treatment	N/A	N/A	N/A	Primary	N/A	N/A	43 ± 7 months after ACL injury Swedish version of the ACL-RSI score = 4.9 ± 2.4
Ueda et al., 2024 [117]	N/A	N/A	No second ACL injury group: preinjury Tegner score = 7.5 ± 1.4 Second ACL injury group: preinjury Tegner score = 8.4 ± 1.0	169 (97/72)	No second ACL injury group: 26.6 ± 11.6 Second ACL injury group: 17.5 ± 4.0	No second ACL injury group: BMI = 23.0 ± 2.7 (kg/m²) Second ACL injury group: BMI = 21.7 ± 3.0 (kg/m²)	Surgical treatment	No Second ACL injury group: 14.0 ± 58.7 (months) Second ACL injury group: 3.1 ± 4.1 (months)	ACLR	Hamstring tendon autografts (N = 169)	Primary	N/A	N/A	No Second ACL injury group: 3 months postoperative Japanese version of the ACL-RSI score = 51.1 ± 22.5 Second ACL injury group: 3 months postoperative Japanese version of the ACL-RSI score = 65.7 ± 23.3
**(4) Predicting Post-Return Sport Performance Levels and Physical Activity Levels**														
Albano et al., 2020 [41]	Athlete	Basketball Soccer (n = 49) Volleyball	Recreational level	N = 150 (130/20)	RTS group: 28.7 ± 7.1 Non-RTS group: 27.4 ± 7.2	RTS group: Height = 173 ± 7.8 (cm) Weight = 81.7 ± 14.4 (kg); BMI = 27.8 ± 7.4 (kg/m²) Non-RTS group: Height = 173.4 ± 7.3 (cm) Weight = 81.7 ± 15.3 (kg) BMI = 27.1 ± 4.1 (kg/m²)	Surgical treatment	RTS group: 14.2 ± 31.6 month Non-RTS group: 17.4 ± 37.9 month	ACLR	Hamstring tendon (n = 129) Patellar tendon (n = 21)	N/A	N/A	N/A	RTS group: 32 ± 29.9 months postoperatively ACL-RSI score = 52.4 ± 17.6 Non-RTS group: 17 ± 18.7 months postoperatively ACL-RSI score = 40.3 ± 18.6 RTS at the preinjury level group: ACL-RSI score = 70.6 ± 19.1 No RTS at the preinjury level group: ACL-RSI score = 44.1 ± 16.6
Ardern et al., 2013 [118]	Athlete	Australian football (n = 67) Netball (n = 29) Soccer (n = 27) Basketball (n = 21)	Recreational level (n = 54) Competitive level (n = 133)	N = 178 (115/63)	27.3 ± 8.7	N/A	Surgical treatment	29.4 ± 65.9 weeks	ACLR	Doubled semitendinosus or doubled gracilis tendon autograft (n = 170) Others (n = 17)	Primary (n = 167) Revision ACLR (n = 11)	In sports (n = 182) Out of sports (n = 5)	N/A	4 months postoperatively RTS group: ACL-RSI score = 57.3 ± 20.3 Non-RTS group: ACL-RSI score = 40.4 ± 17.1
Beischer et al., 2023 [119]	N/A	Football (n = 69) Floorball Handball (n = 23) Alphine skiing/Snowboard Cycling/spinning Running/jogging (n = 23) Strength training (n = 23) Others	Preinjury Tegner score: ≤ 5 (n = 58) 6 (n = 31) 7 (n = 56) 8 (n = 51) 9 (n = 57) 10 (n = 19)	N = 272 (131/141)	Median (range) = 28.9 (16.5–65.3)	BMI: median (range) = 23.7 (16.7–34.0) (kg/m²)	Surgical treatment	Median (range) = 0.4 (0.0–20.6) year	ACLR	Hamstring tendon autograft (n = 215) Bone–patella bone–tendon autograft (n = 19) Allograft (n = 6) Missing (n = 32)	Primary	N/A	N/A	3- to 5-year follow-ups Maintained PA group: Swedish version of the ACL-RSI score: Median (range) = 98 (26–120) Not maintained PA group: Swedish version of the ACL-RSI score: Median (range) = 79 (21–16)
Faleide et al., 2021a [25]	Athlete	Soccer (n = 51) Handball (n = 13) Alpine skiing (n = 6) Cross-country/mountain running (n = 6)	Preinjury IKDC level: Level I (n = 71) Level II (n = 17) Level III (n = 15) Competitive level: Elite level (n = 5) Medium/high competitive level (n = 29) Low competitive level (n = 37) Recreational level (n = 32)	N = 103 (55/48)	28.7 ± 10	N/A	Surgical treatment	Median (IRQ) = 8 (11) months	ACLR	Hamstring tendon autograft (n = 41) Bone–patellar tendon–bone autograft (n = 62)	N/A	N/A	N/A	10.4 ± 1.3 months postoperatively All Patients: Norwegian version of the ACL-RSI score = 55.8 ± 22.4 RTS group: Norwegian version of the ACL-RSI score = 63.5 ± 20.8 Non-RTS group: Norwegian version of the ACL-RSI score = 50.3 ± 22.0
Garra et al., 2024 [120]	N/A	Soccer (n = 24) Basketball (n = 20) Running (n = 16) Skiing (n = 7) Football (n = 5) Other (n = 41)	ACLR and Segond fracture group Preinjury Tegner score = 6.7 ± 1.8 ACLR group Preinjury Tegner score = 7.0 ± 2.0	N = 120 (78/42)	ACLR and Segond fracture group: 32.2 ± 13.6 ACLR group: 33.0 ± 12.7	ACLR and Segond fracture group BMI = 25.3 ± 4.0 (kg/m²) ACLR group BMI = 25.9 ± 4.2 (kg/m²)	Surgical treatment	N/A	ACLR	Bone-patellar tendon-bone autograft (n = 63) Hamstring autograft (n = 21) Quadriceps autograft (n = 18) Allograft (n = 18)	Primary	N/A	N/A	ACLR and Segond fracture group: 5.64 ± 2.38 years postoperatively Short version of the ACL-RSI score = 62.2 ± 25.4 ACLR group: 5.82 ± 2.52 years postoperatively Short version of the ACL-RSI score = 56.6 ± 25.4
Hopper et al., 2024 [121]	N/A	Soccer (n = 27) Skiing (n = 10) Lacrosse (n = 15) Basketball (n = 27) Football (n = 18) Volleyball (n = 9)	Preinjury Tegner score: median (range) = 9 (1 –9)	N = 164 (82/82)	22.5 ± 8.9	Height = 171.6 ± 11.0 (cm) Weight = 77.4 ± 18.6 (kg)	Surgical treatment	N/A	ACLR	Quadriceps (n = 3) Hamstring (n = 26) Patellar (n = 124)	Primary	N/A	Contact injury (n = 39) Non-contact injury (n = 104)	8.56 ± 3.35 months postoperatively Male: ACL-RSI score = 70.9 ± 24.1 Female: ACL-RSI score = 70.3 ± 24.6
Kitaguchi et al., 2020 [122]	Athlete	N/A	Preinjury Tegner score: median (IQR) = 9 (8–9) Competitive level (n = 124)	N = 124 (50/74)	17.0 ± 2.7	N/A	Surgical treatment	3.3 ± 4.2 months	ACLR	Bone-patellar tendon-bone (n = 38) Autologous semitendinosus tendon (n = 86)	Primary	N/A	Contact injury (n = 86) Non-contact injury (n = 38)	6 months postoperatively Total sample: Japanese version of the ACL-RSI score = 59.8 ± 19.6 RTS group: Japanese the version of ACL-RSI score = 63.4 ± 18.7 Non-RTS group: Japanese version of the ACL-RSI score = 43.7 ± 15.4
Rossi et al., 2022 [123]	Athlete Non-athlete	Rugby (n = 9) Soccer (n = 4) Martial arts (n = 4) Basketball (n = 2) Volleyball (n = 1) Rowing (n = 1) Gym (n = 1)	Competitive level (n = 84) Recreational level (n = 20)	N = 104 (90/14)	RTS group: median (IRQ) = 23 (20-32) Non-RTS group: median (IRQ) = 25.5 (22-32)	N/A	Surgical treatment	N/A	Latarjet procedure (n = 50) ABR (n = 54)	N/A	Primary	N/A	N/A	RTS group: median (IQR) = 19.5 (15–26) months postoperatively SI-RSI score: median (IQR) = 38.5 (35–41) Non-RTS group: median (IQR) = 21.5 (17–25) months postoperatively SI-RSI score: median (IQR) = 65 (57–80)
Sadeqi et al., 2018 [124]	Athlete	Pivot with contact (n = 434) Pivot without contact (n = 138) Without pivot (n = 109)	Professional level (n = 26) Competitive level (n = 272) Regular leisure (n = 294) Occasional leisure (n = 89)	N = 681 (467/214)	30.2 ± 9.5	N/A	Surgical treatment	N/A	ACLR	Hamstring tendon (n = 600) Bone–patellar tendon–bone (n = 67) Combined fasciae latae (n = 14)	Primary (n = 611) Revision ACLR (n = 70)	In sports (n = 604) Out of sports (n = 77)	N/A	6 months postoperatively RTS group: French version of the ACL-RSI score = 61.8 ± 21.2 Non-RTS group: French version of the ACL-RSI score = 51.5 ± 22.9 1 year postoperatively RTS group: French version of the ACL-RSI score = 68.1 ± 22.8 Non-RTS group: French version of the ACL-RSI score = 53.3 ± 25.4 2 years postoperatively RTS group: French version of the ACL-RSI score = 70.4 ± 22.8 Non-RTS group: French version of the ACL-RSI score = 49.8 ± 26.2
Slagers et al., 2021a [125]	Athlete	N/A	Recreational level	119 (76/43)	44.0 ± 14	Height = 179.6 ± 9.3 (cm) BMI = 26.3 ± 4.1 (kg/m²)	N/A	Symptom duration 39.9 ± 42.8 months	N/A	N/A	N/A	N/A	N/A	Symptom duration: 39.9 ± 42.8 months Dutch version of the I-PRRS score = 39.2 ± 12.3
Slagers et al., 2021b [126]	Athlete (n = 45) Non-athlete (n = 5)	Cyclic (n = 11) Acyclic (n = 21) Cyclic and acyclic (n = 13) None (n = 5)	Recreational level (n = 38) Regional level (n = 5) (Inter)national level (n = 2) None (n = 5)	N = 50 (34/16)	42.6 ± 12.3	Height = 179.6 ± 11.6 (cm) BMI = 25.5 ± 3.2 (kg/m²)	Surgical treatment (n = 14) Conservative treatment (n = 36)	N/A	N/A	N/A	N/A	In sports (n = 44) Out of sports (n = 6)	N/A	3 months after ATR Dutch version of the I-PRRS: median (IQR) score = 40.0 (23) 6 months after ATR Dutch version of the I-PRRS: median (IQR) score = 45.0 (24) 12 months after ATR Dutch version of the I-PRRS: median (IQR) score = 55.0 (13)
Stigert et al., 2023 [127]	N/A	N/A	Preinjury Tegner score: ≤ 5 (n = 44) 6 (n = 21) 7 (n = 32) 8 (n = 33) 9 (n = 34) 10 (n = 9) Physical activity level: Median (range) = 345 (0–540) min/week	173 (92/81)	Physically active group: 30.8 ± 11.2 Physically inactive group: 32.2 ± 12.1	Total sample BMI = 24.2 ± 2.6 (kg/m²) Physically active group BMI = 24.1 ± 2.4 (kg/m²) Physically inactive group BMI = 25.0 ± 3.2 (kg/m²)	Surgical treatment	N/A	ACLR	N/A	Primary	N/A	N/A	5–8 years postoperatively 21 ≤ ACL-RSI score < 56 (n = 12) 56 ≤ ACL-RSI score < 77 (n = 3) 77 ≤ ACL-RSI score < 119 (n = 6)
Suzuki et al., 2023 [128]	N/A	N/A	Preoperative Tegner score: RTS group A (RTS at or above preinjury level): 7.1 ± 0.8 RTS group B (RTS below preinjury level): 7.0 ± 0.7 Non-RTS group: 6.0 ± 0.0 12 months postoperative Tegner score: RTS group A: 7.1 ± 0.8 RTS group B: 7.0 ± 0.7 Non-RTS group: 2.3 ± 0.4	47 (19/28)	RTS group A: 17.6 ± 4.6 RTS group B: 26.6 ± 11.2 Non-RTS group: 43.5 ± 12.2	N/A	Surgical treatment	N/A	ACLR	Hamstring tendon autografts (N = 47)	Primary	N/A	N/A	RTS group A: 6 months postoperative Japanese version of the ACL-RSI score = 79.8 ± 16.9 12 months postoperative Japanese version of the ACL-RSI score = 88.9 ± 12.6 RTS group B: 6 months postoperative Japanese version of the ACL-RSI score = 59.9 ± 18.0 12 months postoperative Japanese version of the ACL-RSI score = 66.9 ± 18.6 Non-RTS group: 6 months postoperative Japanese version of the ACL-RSI score = 19.8 ± 8.6 12 months postoperative Japanese version of the ACL-RSI score = 32.9 ± 30.6

*Note. *If not otherwise specified, the age of the participants is presented as mean ± SD; IQR = interquartile range; N/A = No data or not applicable; BMI = Body Mass Index; RTS = Return to Sport; ACLR = Anterior Cruciate Ligament Reconstruction; ACL-RSI = Anterior Cruciate Ligament-Return to Sport after Injury; IKDC= International Knee Documentation Committee; KOOS = Knee Injury and Osteoarthritis Outcome Score; BEAR = Bridge-enhanced ACL restoration; I-PRRS = The Injury Psychological Readiness to Return to Sport; SI-RSI = Shoulder Instability-Return to Sport after Injury; CSAS = Cincinnati Sports Activity Scale; ABR = arthroscopic Bankart repair; MPFL-RSI = medial patellofemoral ligament-return to sport after injury; PA = physical activity; MPFLR = medial patellofemoral ligament reconstruction; ATR = Achilles tendon rupture; SLAP = superior-labrum anterior-posterior.

^*^ The Tegner Activity Scale is a tool used to assess an individual’s activity level before and after sports injuries or surgeries (Briggs et al., 2009).

^†^The IKDC (International Knee Documentation Committee) classification system is primarily used to assess knee function and activity level following an injury (Higgins et al., 2007).

^‡^The Marx Activity Score is a tool used to assess an individual’s activity level and function following a knee injury or surgery (Marx et al., 2001).

^#^The Cincinnati Sports Activity Scale is used to assess the level of sports activity of a patient prior to the injury (Barber-Westin & Noyes, 1999).

The methodological quality appraisal used the Mixed Methods Appraisal Tool (MMAT) to evaluate the methodological quality of the included studies [131]. The MMAT covers five common study designs. In this review, we specifically applied this tool to the quantitative (non-randomised) and quantitative (randomized controlled trials) study designs. For these two design categories, the MMAT includes two screening questions (i.e., “Are there clear research questions?” and “Do the collected data allow addressing the research questions?”) and five criteria for each to assess the methodological quality of the included quantitative research. Each criterion is rated on a scale of “yes,” “no,” and “can’t tell”.

## Results

**Fig. 1:**
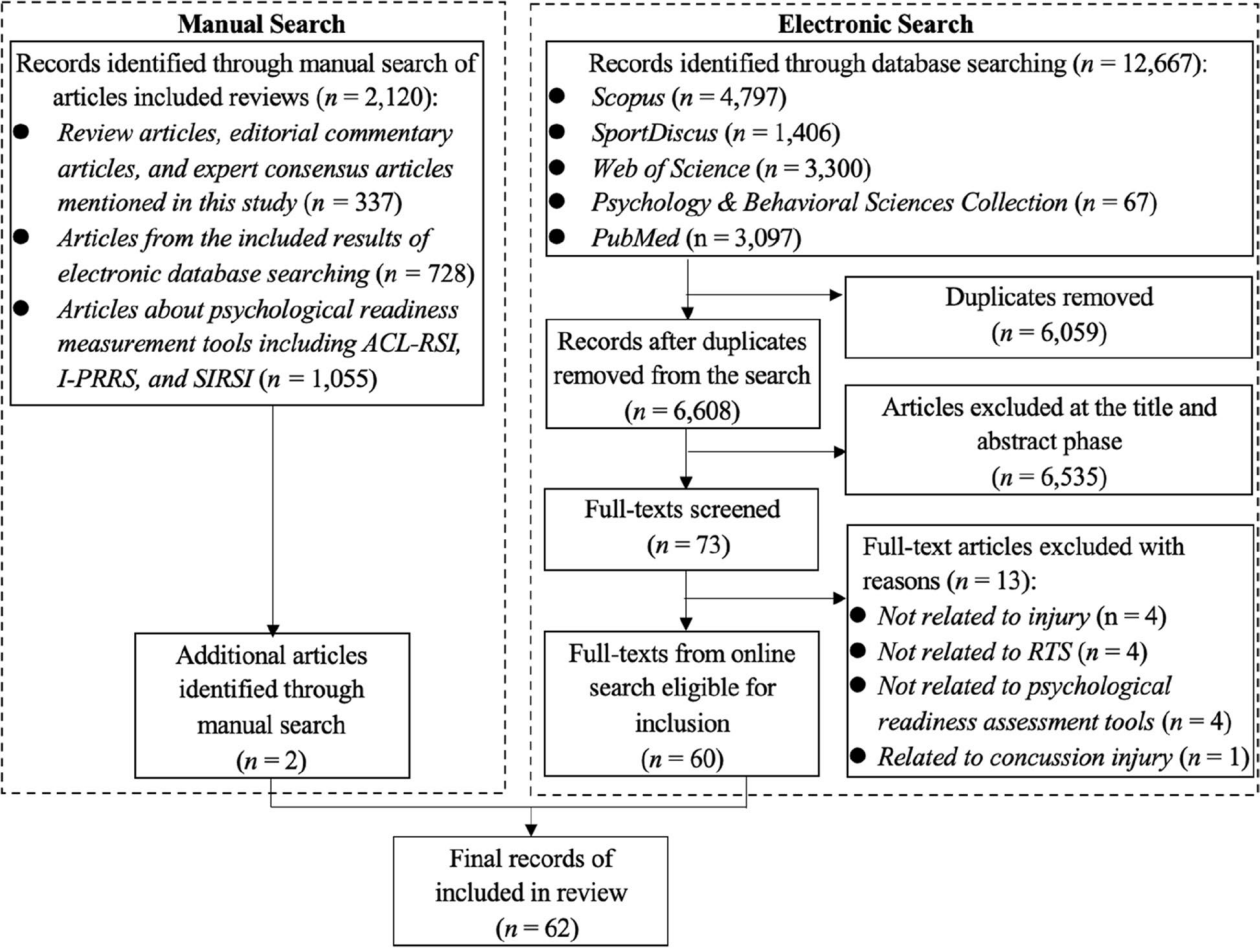
The PRISMA flow diagram

The electronic search yielded a total of 12,667 articles. Following the screening process, 60 articles were included (see Fig. 1). A manual search yielded two more articles [81, 96]; therefore, a total of 62 articles were included in this review. Based on the different predictive role of psychological readiness assessments, these included papers were categorised into four domains: (1) predicting physical recovery outcomes (*n* = 24), (2) predicting return to sport practices (*n* = 18), (3) predicting quality of life-related to injury and reinjury rates (*n* = 7), and (4) predicting post-return sport performance levels and physical activity levels (*n* = 13).

1. Predicting physical recovery outcomes

A total of 24 studies demonstrated that psychological readiness assessment for RTS can predict physical recovery outcomes, including both objective measures and self-reported outcomes [70–93]. Of these studies, 23 utilised the ACL-RSI scale to examine the correlation between psychological readiness and physical recovery outcomes in individuals with ACL injuries, while one study used the SI-RSI scale [81]. The findings consistently indicated that psychological readiness can predict various aspects of physical recovery, such as muscle unit characteristics in the thigh [87], hamstring/quadriceps strength and single-leg hop distance [75, 77, 80, 82, 86, 89], the range of motion in the knee and hip joints [79, 84], the weight ratio of knee strength [71], knee symptoms [72], neuromuscular asymmetry [73], single-leg drop landing knee excursion [76], knee valgus motion [74] and knee joint movement symmetry [85, 88, 91, 92]. In addition to objective recovery measures, psychological readiness assessment can also predict self-reported assessments of physical function, including self-perceived knee performance [78, 83, 90], subjective running ability [70], subjective shoulder function [81], and self-perceived physical capability standards [93].

(2) Predicting return to sport practices

A total of 18 studies explored the relationship between psychological readiness for RTS and RTS practices [33, 94–110]. For example, using the ACL-RSI scale, positive associations were observed in cross-sectional surveys, a prospective cohort study, and a retrospective case–control study conducted at 6 months [94, 105], 8 and 12 months [95, 97, 108], 2 years [98, 103, 107, 110], and even 5 years [104] post-ACLR. Furthermore, psychological readiness scores assessed at 3 and 6 months post-ACL surgery can predict the Likelihood of RTS at 12 months post-surgery [101, 106, 109].

Beyond ACLR, psychological readiness assessed with the SI-RSI scale has been Linked to RTS outcomes following shoulder surgeries, such as arthroscopic Bankart repair and Latarjet, with positive associations noted at 8 months [96], 2 years [100], 40 months [99], and even 5 years [33]. In addition, the adapted ACL-RSI scale, referred to as the Medial Patellofemoral Ligament–Return to Sport after Injury (MPFL-RSI) scale, has been shown to predict RTS outcomes after the Medial Patellofemoral Ligament reconstruction [102].

(3) Predicting quality of life-related to injury and reinjury rates

Seven studies examined whether psychological readiness for RTS can predict quality of life related to injury and reinjury rates [111–117]. Three studies found that psychological readiness assessments can predict quality of life related to the injury [112, 114, 116]. Another four studies found that psychological readiness assessments can predict reinjury rates post-RTS [111, 113, 115, 117].

(4) Predicting post-return sport performance levels and physical activity levels

A total of 13 studies investigated the relationship between psychological readiness for RTS and post-RTS performance levels and physical activity levels [25, 41, 118–128]. Among these, six studies using the ACL-RSI found that psychological readiness assessment can predict sports levels after RTS [25, 41, 118, 122, 124, 128]. Additionally, three studies using the SI-RSI and I-PRRS found that psychological readiness assessment can predict sports levels after RTS [123, 125, 126]. In addition, Beischer et al. [119], Garra et al. [120], Hopper et al. [121], and Stigert et al. [127] found that psychological readiness assessment can predict physical activity levels at 8 months, 12 months, 18 months, and 6 years post-ACLR, respectively.

## Assessment of the quality/rigor of included studies

The methodological quality appraisal is presented in Table 3. No studies were excluded due to methodological quality issues, which aligns with the recommendations of Hong et al. [131].

**Table 3:** MMAT quality appraisal of studies (Alphabetical Order)

**Author(s)**	Q1^*^	**Q2**^†^	**Quantitative (non-randomized)**					**Quantitative (randomized controlled trials)**					**Comments**
Aizawa et al., 2020 [70]	✓	✓	✓	✓	✓	×	✓						• The study did not account for confounders such as injury context and injury mechanism.
Aizawa et al., 2022 [71]	✓	✓	✓	✓	✓	×	✓						• The study did not account for confounders such as injury context.
Aizawa et al., 2024 [94]	✓	✓	✓	✓	✓	×	✓						• The study did not account for confounders such as injury context.
Albano et al., 2020 [41]	✓	✓	✓	✓	✓	×	✓						• The study did not account for confounders such as injury context and surgical history.
Almeida et al., 2023 [111]	✓	✓	✓	✓	✓	×	✓						• The study did not account for confounders such as surgical history, injury context, and injury mechanism.
Ardern et al., 2013 [118]	✓	✓	✓	✓	✓	×	✓						• The study did not account for confounders such as anthropometric measurements and injury mechanisms.
Azevedo Tavares et al., 2023 [112]	✓	✓	✓	✓	✓	×	✓						• The study did not account for confounders such as surgical history, injury context, injury mechanism, and time duration from injury to surgery.
Baez et al., 2023 [72]	✓	✓	✓	✓	✓	×	✓						• The study did not account for confounders such as injury context and time duration from injury to surgery.
Beischer et al., 2019 [95]	✓	✓	✓	×	×	×	✓						• The validity and reliability of the self-created 3-item motivation measurement tool were not validated. ➢ The actual sample size in the subgroups was insufficient to meet the requirements of the sample size analysis. ♢ The study did not account for confounders such as graft type, surgical history, injury context, and injury mechanism.
Beischer et al., 2023 [119]	✓	✓	–	✓	✓	×	✓						• The ACL-RSI questionnaire was introduced 1.5 years after the start of the project. ➢ The study did not account for confounders such as injury context and injury mechanism.
Bohu et al., 2021 [96]	✓	✓	–	✓	✓	✓	✓						• Although the study found no significant association between psychological readiness and sport type, it is important to note that sports were categorized into only four types (e.g., overhead contact, overhead non-contact, non-overhead contact, non-overhead non-contact) in the study.
Borawski et al., 2024 [73]	✓	✓	✓	✓	✓	×	✓						• The study did not account for confounders such as time duration from injury to surgery, surgical history, injury context, and injury mechanism. ➢ The study reported the graft type used for ACLR patients; however, in the limitations section, the authors noted that they did not provide graft type information.
Colasanti et al., 2023 [33]	✓	✓	✓	✓	✓	×	✓						• The study did not account for confounders such as anthropometric measurements, time duration from injury to surgery, surgical history, injury context, and injury mechanism.
Correa et al., 2023 [74]	✓	✓	×	✓	✓	×	✓						• The study included only male athletes. ➢ The study did not account for confounders such as injury context and injury mechanism.
Cronstrom et al., 2023b [75]	✓	✓	×	✓	✓	×	✓						• Approximately 75% of eligible patients did not respond to the invitation to participate in the study, which may have resulted in an insufficient representation of the target population and introduced selection bias. ➢ The study did not account for confounders such as injury context.
Dombrowski et al., 2024 [76]	✓	✓	✓	✓	✓	×	✓						• The study did not account for confounders such as time duration from injury to surgery, surgical history, injury context, injury mechanism.
Erickson et al., 2022 [77]	✓	✓	✓	✓	✓	×	✓						• The study did not account for confounders such as injury context and injury mechanism.
Faleide et al., 2021a [25]	✓	✓	✓	✓	✓	×	✓						• The study did not account for confounders such as anthropometric measurements, surgical history, injury context, and injury mechanism.
Faleide et al., 2021b [97]	✓	✓	✓	✓	✓	×	✓						• The study did not account for confounders such as anthropometric measurements, time duration from injury to surgery, injury context, and injury mechanism.
Fones et al., 2020 [78]	✓	✓	×	✓	✓	×	✓						• Approximately 75% of eligible patients did not respond to the invitation, indicating the presence of selection bias. ➢ The limitations of a retrospective design may lead to insufficient control and analysis of confounders, thereby affecting the interpretation of the results. ♢ The study did not account for confounders such as injury context and injury mechanism.
Fältström et al., 2016 [98]	✓	✓	×	×	✓	×	✓						• The study included only female athletes and the response rate was 60%. ➢ Motivation assessment is subject to recall bias. ♢ The study did not account for confounders such as injury context.
Garra et al., 2024 [120]	✓	✓	×	✓	✓	×	✓						• The average age of the sample in this study is older compared to the sample in previous studies. ➢ The study did not account for confounders such as time duration from injury to surgery, injury context, and injury mechanism.
Hart et al., 2020 [79]	✓	✓	✓	×	✓	×	✓						• The visual analog scale (VAS) used to assess knee confidence during performance-based functional tasks has not been validated. ➢ The study did not account for confounders such as time duration from injury to surgery, injury context, and injury mechanism.
Hopper et al., 2024 [121]	✓	✓	✓	✓	✓	×	✓						• The study did not account for confounders such as time duration from injury to surgery and injury context.
Hurley et al., 2022a [81]	✓	✓	✓	×	✓	×	✓						• Uncertain what specific aspect of satisfaction is being measured by the 1-item 5-point Likert scale (ranging from “very dissatisfied” to “very satisfied”). ➢ The study did not account for confounders such as anthropometric measurements, time duration from injury to surgery, surgical history, injury context, and injury mechanism.
Hurley et al., 2022b [99]	✓	✓	×	✓	✓	×	✓						• The study included only male athletes. ➢ The study did not account for confounders such as anthropometric measurements, time duration from injury to surgery, injury context, and injury mechanism.
Hurley et al., 2022c [100]	✓	✓	✓	✓	✓	×	✓						• The study did not account for confounders such as anthropometric measurements, time duration from injury to surgery, injury context, and injury mechanism.
Högberg et al., 2023 [80]	✓	✓	✓	×	✓	×	✓						• Inadequate warm-up and familiarization with the seated isokinetic Biodex test may have affected the measurement results of absolute peak torque. ➢ The study did not account for confounders such as surgical history, injury context, and injury mechanism.
Kitaguchi et al., 2020 [122]	✓	✓	✓	✓	×	×	✓						• Due to follow-up loss, social reasons, and second ACL injuries, 46 athletes were excluded, resulting in a final sample size that was slightly smaller than the sample size calculated by power analysis. ➢ Whether patients have returned to their preinjury level of sports activities is based on self-report, which means the measurement may rely on the patient’s subjective judgment rather than objective data or validated tools. This could lead to potential bias in the measurement results. ♢ The study did not account for confounders such as anthropometric measurements and injury context.
Langford et al., 2009 [101]	✓	✓	✓	✓	✓	×	✓						• The study did not account for confounders such as anthropometric measurements, injury context, and injury mechanism.
Legnani et al., 2023 [82]	✓	✓	–	–	✓	×	✓						• At the time of injury, there were a total of 49 patients (21 in contact sports and 28 in non-contact sports), but the total number of participants in this study was 31. ➢ In Sect. 3.2, the ACL-RSI scores described in the text and the table are inconsistent. ♢ The study did not account for confounders such as injury context.
Li et al., 2023 [102]	✓	✓	✓	✓	✓	×	✓						• The study did not account for confounders such as anthropometric measurements, time duration from injury to surgery, injury context, and injury mechanism.
Liew et al., 2022 [103]	✓	✓	✓	✓	✓	×	✓						• The study did not account for confounders such as anthropometric measurements, time duration from injury to surgery, injury context, and injury mechanism.
Manara et al., 2022 [104]	✓	✓	✓	✓	✓	×	✓						• The study did not account for confounders such as injury mechanism.
McPherson et al., 2019a [113]	✓	✓	✓	✓	✓	×	✓						• The study did not account for confounders such as time duration from injury to surgery, injury context, and injury mechanism.
Milewski et al., 2023 [83]	✓	✓	×	✓	✓	×	✓						• The study has a small sample size. ➢ The graft type was not randomized and rather was based on preferences of the orthopaedic surgeon and patient/family. ♢ The study did not account for confounders such as time duration from injury to surgery and injury mechanism.
Müller et al., 2015 [105]	✓	✓	✓	✓	✓	×	✓						• The study did not account for confounders such as surgical history and injury mechanism.
Nagelli et al., 2019 [84]	✓	✓	×	✓	✓	×	✓						• The study has a small sample size (*N* = 18). ➢ The study did not account for confounders such as time duration from injury to surgery, surgical history, injury context, and injury mechanism.
Olds and Webster, 2021 [114]	✓	✓	×	✓	✓	×	✓						• The study has a small sample size (*N* = 80) and a limited number of female participants (*n* = 18). ➢ The study did not account for confounders such as anthropometric measurements, time duration from injury to surgery, surgical history, injury context, and injury mechanism.
Pasqualini et al., 2024 [115]	✓	✓	✓	✓	✓	×	✓						• The study did not account for confounders such as anthropometric measurements, time duration from injury to surgery, and injury context.
Peebles et al., 2021 [85]	✓	✓	×	✓	✓	×	✓						• The study has a small sample size (*N* = 38). ➢ The study did not account for confounders such as time duration from injury to surgery and injury mechanism.
Rossi et al., 2022 [123]	✓	✓	✓	✓	✓	×	✓						• The study did not account for confounders such as anthropometric measurements, time duration from injury to surgery, injury context, and injury mechanism.
Sadeqi et al., 2018 [124]	✓	✓	✓	✓	✓	×	✓						• The study did not account for confounders such as anthropometric measurements, time duration from injury to surgery, and injury mechanism.
Sanborn et al., 2022 [86]	✓	✓						✓	✓	✓	✓	✓	• This study meets all the criteria for MMAT assessment.
Schilaty et al., 2023 [87]	✓	✓	✓	✓	✓	×	✓						• The study did not account for confounders such as injury context.
Slagers et al., 2021a [125]	✓	✓	✓	✓	✓	×	✓						• The study did not account for confounders such as treatment methods, surgical history, injury context, and injury mechanism.
Slagers et al., 2021b [126]	✓	✓	×	✓	✓	×	✓						• The study has a relatively small sample size (*N* = 50). ➢ The study did not adjust multiple linear regression analysis for body mass index and comorbidities. ♢ The study did not account for confounders such as time duration from injury to surgery, surgical history, and injury mechanism.
Slater et al., 2023 [106]	✓	✓	✓	✓	✓	✓	✓						• This study meets all the criteria for MMAT assessment.
Sonesson et al., 2021 [116]	✓	✓	✓	✓	✓	✓	✓						• This study meets all the criteria for MMAT assessment.
Stigert et al., 2023 [127]	✓	✓	✓	✓	✓	×	✓						• The study did not account for confounders such as time duration from injury to surgery, injury context, and injury mechanism.
Sugarman et al., 2022 [88]	✓	✓	✓	✓	✓	×	✓						• The study did not account for confounders such as time duration from injury to surgery, graft type, injury context, and injury mechanism.
Suzuki et al., 2023 [128]	✓	✓	✓	×	✓	×	✓						• The study did not assess the validity and reliability of the subjective RTS level. ➢ The study did not account for confounders such as anthropometric measurements, time duration from injury to surgery, injury context, and injury mechanism.
Toale et al., 2021 [107]	✓	✓	✓	✓	×	×	✓						• The study reported that the ACL-RSI completion rate was less than 50% at the 2-year follow-up. ➢ The study did not account for confounders such as time duration from injury to surgery and injury context.
Ueda et al., 2022 [89]	✓	✓	✓	✓	✓	×	✓						• The study did not account for confounders such as injury context and injury mechanism.
Ueda et al., 2024 [117]	✓	✓	✓	✓	✓	×	✓						• The study did not account for confounders such as injury context and injury mechanism.
Webster and Feller, 2020 [90]	✓	✓	✓	✓	✓	×	✓						• The study did not account for confounders such as anthropometric measurements, time duration from injury to surgery, injury context, and injury mechanism.
Webster and Feller, 2022 [109]	✓	✓	✓	✓	✓	×	✓						• The study did not account for confounders such as anthropometric measurements, time duration from injury to surgery, and injury mechanism.
Webster et al., 2018 [91]	✓	✓	✓	✓	✓	×	✓						• The study did not account for confounders such as anthropometric measurements, injury context, and injury mechanism.
Webster et al., 2022 [110]	✓	✓	×	✓	✓	×	✓						• The study included only male athletes. ➢ The study did not account for confounders such as anthropometric measurements.
Zarzycki et al., 2018 [92]	✓	✓	✓	✓	✓	×	✓						• The study did not account for confounders such as time duration from injury to surgery, graft type, injury context, and injury mechanism.
Zwolski et al., 2023 [93]	✓	✓	✓	✓	✓	×	✓						• The study did not account for confounders such as time duration from injury to surgery, injury context, and injury mechanism.
van Haren et al., 2023 [108]	✓	✓	✓	✓	✓	×	✓						• Due to the involvement of different physical therapists and the fact that all baseline measurements were conducted by each participant’s own physical therapist, there may be some measurement bias. ➢ The study did not account for confounders such as time duration from injury to surgery, injury context, and injury mechanism.

^*^Q1 = screening questions 1: Are there clear research questions?

^†^Q2 = screening questions 2: Do the collected data allow addressing the research questions?

*MMAT* Mixed Methods Appraisal Tool; ‘✓’ means that the criterion is met, ‘ × ’ indicates that the criterion is not met, and ‘–’ means that there is not enough information in the paper to judge if the criterion is met or not. Additionally, the assessment columns for three study designs (i.e., qualitative, quantitative descriptive, and mixed methods) have been removed because these types of study designs were not utilized in this review study. Five criteria for the Quantitative (non-randomized) studies are as follows: 1. Are the participants representative of the target population? 2. Are the measurements appropriate in relation to both the outcome and the intervention (or exposure)? 3. Are the outcome data complete? 4. Are the confounders addressed in the study design and analysis? 5. Was the intervention administered (or exposure occurred) as intended during the study period? Five criteria for the Quantitative (non-randomized) studies are as follows: 1. Is randomization appropriately performed? 2. Are the groups comparable at baseline? 3. Are there complete outcome data? 4. Are outcome assessors blinded to the intervention provided? 5 Did the participants adhere to the assigned intervention?

## Discussion

This review aims to consolidate evidence regarding the predictive role of psychological readiness assessments for RTS. A total of 62 studies were included through manual and electronic database searches. These studies, employing various research designs (e.g., cross-sectional, prospective cohort, and retrospective cohort), examined associations between psychological readiness assessments and RTS outcomes after injury. Based on these findings, this review addresses: (1) validity of existing psychological readiness assessments, (2) consideration of confounding variables, (3) recommendations for athletes with low psychological readiness scores, and (4) cautions when applying psychological readiness measures.

1. Validity of existing psychological readiness assessments

This review draws conclusions from studies utilising the ACL-RSI scale [31], I-PRRS scale [32], MPFL-RSI scale, SLAP-RSI scale [33], and SI-RSI scale [34]. The I-PRRS scale can be used to measure psychological readiness for RTS in athletes with different types of injuries, but it focuses solely on confidence levels [17]. The ACL-RSI includes three dimensions: emotions, confidence in performance, and risk appraisal [31]. The SLAP-RSI, MPFL-RSI, and SI-RSI are all adapted from the ACL-RSI. However, just as the ACL-RSI is specifically designed for ACL injuries, the SLAP-RSI, MPFL-RSI, and SI-RSI are limited to specific injuries (e.g., shoulder injury and knee injury). Moreover, the ACL-RSI scale’s emotions dimension includes two items related to fear of reinjury (“Are you fearful of re-injuring your knee by playing your sport?” and “Are you afraid of accidentally injuring your knee by playing your sport?”). However, Olds and Webster [114] argued that it may not fully capture the fear dimension in psychological readiness before RTS, as fear of reinjury represents a distinct aspect of psychological readiness compared to other emotions (e.g., nervousness and frustration). Consequently, in psychological readiness assessment practice, researchers have often used additional measures to evaluate the fear of movement/reinjury, such as the Tampa Scale for Kinesiophobia [67], to provide a more comprehensive assessment of athletes’ psychological states [72, 73, 79, 105, 112, 118, 121, 125, 126, 132].

While the Tampa Scale for Kinesiophobia is recommended as a supplementary tool for assessing psychological readiness before RTS [47], it was not specifically designed for athletes [133]. Athletes may exhibit different psychological responses compared to the general population during RTS [134]. Furthermore, the Tampa Scale for Kinesiophobia is primarily used to identify early signs of injury-related fear during rehabilitation [134]. Given these considerations, Dover and Amar [134] argued that it may not be suitable for evaluating athletes’ psychological states at the RTS stage. Therefore, future studies should use fear assessment scales specifically developed for athletes and the RTS phase, such as the Athlete Fear Avoidance Questionnaire [134] and the Fear of RTS Scale [135]. These efforts may contribute to more accurately capturing the psychological states experienced by injured athletes before RTS, addressing the limitations of existing psychological readiness measures that lack the ability to capture the fear dimension.

In addition to fear, Podlog et al. [17] incorporated motivation as a factor in their latest definition of psychological readiness for RTS. Previous research has predominantly focused on assessing the relationship between motivation and physical recovery outcomes [95], RTS practices [98], and athletic performance [126]. However, a recent study has begun investigating the relationship between motivation and psychological readiness [93]. Moreover, Ohji et al. [10] identified that psychological readiness and self-efficacy are independent variables at the RTS stage, with distinct predictive roles for physical recovery outcomes [77, 106]. Slagers et al. [126] also found a positive relationship between psychological readiness and self-efficacy. However, the current popular measures of psychological readiness for RTS (e.g., the ACL-RSI scale [31], I-PRRS scale [32], MPFL-RSI scale, SLAP-RSI scale [33], and SI-RSI scale [34]) do not include motivation or self-efficacy dimensions. Therefore, Webster and Feller [136] recommended combining the ACL-RSI with other psychological assessment tools to provide a more comprehensive evaluation of patients’ psychological states in RTS practice.

Moreover, the use of various psychological questionnaires to capture pre-return psychological states indicates that current mainstream measures of psychological readiness for RTS may have room for improvement [17]. In addition to using existing psychological readiness measures in conjunction with other psychological assessments (e.g., fear, motivation, or self-efficacy assessments) to evaluate the psychological state of injured athletes before RTS, future research could focus on developing more comprehensive psychological readiness measures based on an updated and comprehensive definition of psychological readiness for RTS [17].

(2) Consideration of confounding variables

The included studies indicated that psychological readiness assessment scores tend to increase over time during post-surgery rehabilitation. For example, Legnani et al. [82] reported increases in ACL-RSI scores from pre-surgery to 3–6 months post-surgery. Similarly, Webster and Feller [109] and Sadeqi et al. [124] found increases in ACL-RSI scores between 6 and 12 months post-surgery and between the immediate post-surgery period and 12 months. However, Gauthier et al. [9], who assessed the psychological and physiological recovery status of 303 patients at 8 and 12 months post-ACLR, found significant increases in quadriceps and hamstring strength over time but no changes in psychological readiness scores.

Upon reviewing the summary table, we propose that these conflicting conclusions may be attributed to a third category of variables that influenced the outcomes, commonly referred to as confounding variables or moderators, depending on the study design and analytic approach. These factors can be grouped into five categories: 1) Demographic variables, such as age [65, 76, 86], gender [65, 76, 91], anthropometrics [9, 111], and population type [9]; 2) Injury-related factors, such as mechanism of injury [19]; 3) Sport-related variables, such as type of sport [9], pre-injury physical activity level [91, 121], and pre-injury performance level [86]; 4) Treatment-related factors, including time between injury and surgery [65, 75, 91], surgical procedures [86], surgical history [132], and treatment options [116]; 5) Contextual factors, such as RTS status [95] and measurement timing [77].

For example, Gauthier et al.’s study did not account for injury context or mechanism [9], despite evidence that these factors (e.g., whether the injury occurred during sport or was contact-related) may influence psychological readiness [19, 137]. Additionally, pre-injury physical activity levels, athlete status, and baseline psychological readiness scores were also omitted. These factors have been shown to affect psychological readiness outcomes [9, 91, 121].

While we have grouped these factors as confounding variables for simplicity, we acknowledge that some may actually function as moderators, influencing the strength or direction of the relationship between psychological readiness and RTS outcomes. This distinction depends on how each study conceptualized and analyzed the variable, which varied widely. Thus, future reviews or meta-analyses may consider evaluating how each study addressed this third category of variables statistically.

Given the complexity and potential overlap of these variables, researchers face substantial challenges in controlling for them. As Sheean et al. [52] noted, psychological readiness for RTS remains an underexplored area. To improve the reliability of future findings, we recommend accounting for as many of these variable types as feasible. This includes the use of standardized assessment protocols, careful participant matching (e.g., based on activity level), subgroup analyses, and statistical controls (e.g., including pre-injury activity as a covariate in multivariate models). Addressing these factors can reduce bias and enhance our understanding of the responsiveness and applicability of psychological readiness measures.

(3) Recommendations for athletes with low psychological readiness scores

It is important to acknowledge that some injured athletes may exhibit persistently low psychological readiness scores from pre-surgery through the RTS phase [138]. Research has indicated that even after passing RTS physical tests and receiving medical clearance, their psychological readiness may remain insufficient [93]. Possible reasons for this include ongoing negative psychological responses (e.g., fear, anxiety, and depression) along with lower self-efficacy, which affect the injured athletes’ RTS practice [93, 138]. Additionally, participation in physical training programs has been found ineffective in improving their psychological readiness for RTS [139]. This is because traditional physical rehabilitation programs primarily focus on agility training, core strengthening, and stability training, while neglecting psychological support for injured athletes. However, expert consensus suggests that psychological readiness for RTS is indeed modifiable [140]. For example, psychological interventions, such as mindfulness interventions, can promote psychological adaptation in injured athletes and help them prepare mentally for RTS [129]. Therefore, continuous assessment of psychological readiness from the early post-surgery period to return to performance is crucial [51, 53, 77].

As summarised in this review, psychological readiness assessments can predict physical recovery outcomes and assist practitioners in deciding whether to delay RTS. For athletes scoring poorly on psychological readiness assessments, tailored psychological interventions that consider their sport, activity level, and RTS goals may enhance their psychological readiness [130, 141]. These interventions can include mindfulness interventions [129], internet-based psychological support [142], cognitive–behavioural interventions [143], and imagery training [144]. For example, in a study involving eight ACLR patients who participated in a seven-session telephone-based cognitive–behavioural intervention initiated before surgery and continuing until 8 weeks post-surgery, six participants reported a decrease or significant decrease in their fear of reinjury [143]. Additionally, welcome-back ceremonies organised by coaches and teammates for RTS may represent a promising approach [145]. These efforts may help athletes improve psychological readiness, reduce the risk of premature return, and prevent reinjury [41, 114], while also decreasing the likelihood of reinjury [113].

(4) Cautions when applying psychological readiness measures

Although existing psychological readiness measures such as ACL-RSI and I-PRRS offer recommended threshold scores (e.g., ACL-RSI ≥ 65 [124], I-PRRS ≥ 50 [32]), it is important to note that these thresholds have not been empirically validated for clinical decision-making. The current evidence does not support the use of such cut-off scores to definitively determine an athlete’s psychological readiness to RTS.

Moreover, research suggests that elevated psychological readiness scores are not always indicative of safe or optimal recovery [132]. For instance, Ueda et al. [117] found that higher psychological readiness at 3 months post-ACLR was associated with an increased risk of second ACL injuries within 24 months. Caumeil et al. [146] proposed that excessive confidence might lead to premature RTS before physical readiness is achieved. In such cases, Caumeil et al. [146] recommended further assessments of optimism and self-esteem to identify potentially inflated confidence. Similarly, Evans and Brewer [62] raised concerns about social desirability bias, which may artificially inflate psychological readiness scores and correlate with increased injury risk. These findings underscore the complexity of interpreting high psychological readiness scores. Podlog et al. [17] suggested that clinicians should be cautious in interpreting such scores and communicate the potential risks of premature RTS. Arvinen-Barrow et al. [139] also noted that high motivation may mask underlying somatic anxiety, further complicating readiness judgments.

Zarzycki et al. [132] introduced the notion of a “*sweet spot*” for psychological readiness, an optimal range in which an athlete’s confidence and motivation are high enough to support safe RTS, but not so elevated as to lead to premature return and increased reinjury risk. This concept suggests that both extremely low and excessively high readiness scores may be detrimental. However, the “*sweet spot*” remains largely theoretical, as there is currently limited empirical evidence to establish upper threshold values, standardize how to operationalize the sweet spot, or understand how individual and cultural factors (e.g., personality traits, sports type, and rehabilitation environment) might shape it. Further research is needed to define this range more precisely and explore its practical application in guiding RTS decisions. More importantly, the use of psychological readiness assessments in clinical RTS decision-making requires further validation. Cut-off scores should be interpreted with caution, and the broader application of these measures should remain exploratory until more robust empirical evidence emerges.

## Limitations

While this review offers valuable insights into psychological readiness assessment for RTS, several limitations should be acknowledged. First, although self-report questionnaires are widely used and recommended by researchers to assess athletes’ psychological readiness for RTS following injury [3, 17, 39, 43–59], a primary limitation of these clinician-friendly tools is their vulnerability to biased or deceptive responses [145]. Factors such as social desirability, overconfidence, or internal or external pressure to return may compromise the validity of the self-reported data. To address these concerns, Brewer et al. [147] explored the use of non-transparent assessment methods such as implicit association tests, information processing tasks, and projective testing, that reduce the risk of deliberate response distortion. Additionally, Liu and Noh [145] have proposed the use of objective neurocognitive measures as promising tools for evaluating psychological readiness during the return-to-sport phase. These complementary approaches warrant further development to enhance the reliability and comprehensiveness of psychological readiness assessments beyond traditional self-report instruments.

Second, the review is based on a limited number of studies utilising various psychological readiness measures, such as the ACL-RSI, MPFL-RSI, SLAP-RSI, and SI-RSI. These scales are specific to certain types of injuries, which may restrict the generalisability of the findings across broader injury types and sports. Third, this review is focused only on quantitative measures of psychological readiness. Consequently, qualitative research findings—particularly those reflecting the perspectives of coaches and clinicians on psychological readiness assessments for RTS—have been overlooked. Fourth, this study focused on electronic database searches that exclusively examined the English versions of psychological readiness tools, excluding non-English translations of these scales. Consequently, research published in other languages and non-English translated scales were not included, which may introduce language bias and limit the overall comprehensiveness of the findings.

Finally, this review excluded studies that employed only individual emotional or self-efficacy measures to assess mental states after sports injuries. As noted by Podlog et al. [17] in their state-of-the-art review on psychological readiness to return to sport, instruments such as the Tampa Scale for Kinesiophobia [67], the Re-Injury Anxiety Inventory [68], and the Knee–Self-Efficacy Scale [69] do not explicitly assess the overarching construct of psychological readiness. Accordingly, Podlog et al. [17] categorized these instruments as “injury-relevant measures” rather than as measures of “psychological readiness for return to sport” (e.g., ACL-RSI scale [31] and I-PRRS scale [32]). Nevertheless, these measures do capture important subcomponents of psychological readiness. As a result, the comprehensiveness of this review is limited, as it does not allow for a comparison of the relative merits of these constructs as potential components of psychological readiness after sports injuries.

## Implications and future directions

The findings of this review may enhance practitioners’ understanding of the applications of psychological readiness assessments in RTS. This increased awareness may, in turn, help reduce the challenges associated with inadequate psychological readiness during the RTS phase. Furthermore, this review provides valuable literature support for researchers in designing psychological interventions and developing psychological readiness questionnaires, thus contributing to the advancement of knowledge in sports injury psychology. Specifically, there are three key suggested areas for future research.

1. Considerations for psychological support strategies
  Although this review compiles studies involving various approaches aimed at supporting psychological readiness in injured athletes, there is a notable lack of validated, standardized protocols for addressing psychosocial factors post-injury [64]. To bridge this gap, future research could explore the development and validation of structured psychological support frameworks to assist clinicians, particularly in guiding decisions for athletes presenting with low psychological readiness scores.
  Emerging evidence also points to the promise of technology-facilitated psychological support (e.g., mobile apps, virtual reality, and online platforms) [139, 142], which may improve accessibility and engagement. However, such tools require further empirical testing to establish their effectiveness in rehabilitation contexts.
  Moreover, recognizing that not all strategies are suitable for every athlete [143], future studies may also consider developing tailored approaches based on individual differences, such as injury type, sport-specific demands, psychological profiles, and stages of recovery. Importantly, any move toward standardization should also be mindful of the need for customization, suggesting that future efforts must balance both consistency and individualization in supporting athletes’ psychological recovery.
2. Development of comprehensive psychological readiness scales
  Existing research predominantly uses the ACL-RSI scale within the context of ACL, making it uncertain whether these findings can be generalised to other injury types (e.g., fractures and concussions). For example, Crofts et al. [148] found that concussions impact the psychological readiness of injured athletes, which subsequently influences both the RTS and post-RTS performance [149]. However, since concussions can lead to cognitive impairments [66], the suitability of existing psychological readiness assessment scales, originally developed for injuries to specific body parts, requires further investigation in the context of concussion-related injuries.
  Furthermore, the ACL-RSI scale and its adaptations (e.g., SLAP-RSI scale, MPFL-RSI scale, and SI-RSI scale) are limited to specific injury sites. Although the I-PRRS scale [32] was designed as a psychological readiness scale for all sports injuries, it focuses solely on confidence before RTS and has raised concerns regarding its structural validity [17]. Recent qualitative research has suggested that psychological readiness for RTS encompasses more than confidence, including factors such as passion for the sport, psychological resilience, and social relationships [19, 20]. Therefore, future research should prioritise the development of multidimensional psychological readiness scales that are applicable to various types of injuries.
3. Focus on non-ACL injury populations
  Most of the studies included in this research are related to ACL injuries. ACL injury is an acute injury, and patients often experience immediate emotional responses due to the sudden loss of physical capability. However, for participants with chronic injuries, the emotional responses and psychological readiness for RTS may differ because the pain mechanisms are distinct from those in acute injuries. However, research in this area is relatively scarce. Therefore, future studies could focus on evaluating the effectiveness of RTS psychological readiness scales for populations with non-ACL injuries (e.g., chronic injuries).

## Conclusion

This review synthesized current evidence on the predictive value of psychological readiness assessments in RTS decisions. Findings indicate that tools such as the ACL-RSI are useful in identifying athletes who are more likely to achieve successful RTS. However, their predictive utility may be influenced by unaccounted confounding or moderating variables such as gender, sport type, and pre-injury activity levels. While these tools offer practical value, overreliance on single threshold scores may overlook complexities such as the potential downsides of excessively high readiness scores. Limitations included the exclusion of qualitative studies and relevant psychological measures such as the Tampa Scale of Kinesiophobia. Future research should focus on refining readiness assessments through the integration of objective indicators and standardized protocols, while addressing key contextual variables to improve accuracy and applicability in diverse athletic populations.

## Data Availability

The data that support the findings of this study are available from the corresponding author, upon reasonable request.
